# Development of Functional and Molecular Correlates of Vaccine-Induced Protection for a Model Intracellular Pathogen, *F. tularensis* LVS

**DOI:** 10.1371/journal.ppat.1002494

**Published:** 2012-01-19

**Authors:** Roberto De Pascalis, Alicia Y. Chou, Catharine M. Bosio, Chiung-Yu Huang, Dean A. Follmann, Karen L. Elkins

**Affiliations:** 1 Laboratory of Mycobacterial Diseases and Cellular Immunology, Division of Bacterial, Parasitic and Allergenic Products, Center for Biologics Evaluation and Research, U.S. Food and Drug Administration, Rockville, Maryland, United States of America; 2 Laboratory of Intracellular Parasites, Rocky Mountain Laboratories, NIAID/NIH, Hamilton, Montana, United States of America; 3 Biostatistics Research Branch, Division of Clinical Research, NIAID/NIH, Bethesda, Maryland, United States of America; Yale University School of Medicine, United States of America

## Abstract

In contrast with common human infections for which vaccine efficacy can be evaluated directly in field studies, alternative strategies are needed to evaluate efficacy for slowly developing or sporadic diseases like tularemia. For diseases such as these caused by intracellular bacteria, serological measures of antibodies are generally not predictive. Here, we used vaccines varying in efficacy to explore development of clinically useful correlates of protection for intracellular bacteria, using *Francisella tularensis* as an experimental model. *F. tularensis* is an intracellular bacterium classified as Category A bioterrorism agent which causes tularemia. The primary vaccine candidate in the U.S., called Live Vaccine Strain (LVS), has been the subject of ongoing clinical studies; however, safety and efficacy are not well established, and LVS is not licensed by the U.S. FDA. Using a mouse model, we compared the *in vivo* efficacy of a panel of qualitatively different *Francisella* vaccine candidates, the *in vitro* functional activity of immune lymphocytes derived from vaccinated mice, and relative gene expression in immune lymphocytes. Integrated analyses showed that the hierarchy of protection *in vivo* engendered by qualitatively different vaccines was reflected by the degree of lymphocytes' *in vitro* activity in controlling the intramacrophage growth of *Francisella*. Thus, this assay may be a functional correlate. Further, the strength of protection was significantly related to the degree of up-regulation of expression of a panel of genes in cells recovered from the assay. These included IFN-γ, IL-6, IL-12Rβ2, T-bet, SOCS-1, and IL-18bp. Taken together, the results indicate that an *in vitro* assay that detects control of bacterial growth, and/or a selected panel of mediators, may ultimately be developed to predict the outcome of vaccine efficacy and to complement clinical trials. The overall approach may be applicable to intracellular pathogens in general.

## Author Summary

Diseases such as tuberculosis (caused by *Mycobacterium tuberculosis*) or tularemia (caused by *Francisella tularensis*) result from infections by microbes that live within cells of a person's body. New vaccines are being developed against such intracellular pathogens, but some will be difficult to test, because disease takes a long time to develop (e.g., tuberculosis) or because outbreaks are unpredictable (e.g., tularemia). Usually such infections are controlled by activities of T cells. However, there are no accepted measures of T cell function that reliably predict vaccine-induced protection. We studied two new ways to do so. We used a group of vaccine candidates against tularemia that stimulated good, fair, or poor protection of mice against *Francisella* challenge. We then measured whether *Francisella*–immune cells from vaccinated mice controlled the growth of bacteria inside cells, and/or whether the expression of immune genes in *Francisella*–immune cells was increased. We found that the degree of protection was matched by the degree of the cells' function in controlling intramacrophage bacterial growth. Further, the degree was predicted by relative amounts of gene expression for several immune mediators. Thus the two new options explored here may help predict protection, without waiting for the onset of disease.

## Introduction

Most vaccines against infectious diseases in clinical use today act by stimulating the production of antibodies, which block virus entry, neutralize toxins, or otherwise limit infection through a variety of mechanisms. Measurements of serum antibodies have therefore been applied to predict successful vaccine-induced protection against diseases such as rabies, tetanus, and diphtheria [1]. In contrast, cell-based immune responses provided by T lymphocytes may be more important for control of intracellular pathogens. To date, however, no predictive correlates have been established for any intracellular pathogen. Understanding T cell effector functions that control intracellular infections, and developing clinically useful predictive correlates, would greatly facilitate evaluation of new vaccines for intracellular pathogens of major public health importance such as *Mycobacteria*, *Chlamydia*, *Salmonella*, and *Leishmania*.

To address these questions, we have exploited experimental infection models that use the Live Vaccine Strain (LVS) of *Francisella tularensis*, a Gram-negative intracellular bacterium that causes tularemia. Although the incidence of tularemia in the U.S. is low, *F. tularensis* is a bioterrorism concern due to its high infectivity and mortality rates following pulmonary infection [2]. Antibiotics are effective, but difficulties with diagnosis and with prompt treatment make developing vaccines a priority [3], [4]. However, the sporadic nature of disease likely means that vaccine field trials for efficacy are impractical.

The use of live attenuated Type B *Francisella* strains as vaccines in the former U.S.S.R. during and after World War II had clear impact on the epidemiology of disease [5]. Successful vaccination of humans using attenuated bacteria has been mimicked experimentally; rabbits, guinea pigs, rats, and mice are all either natural hosts or are susceptible to *Francisella*, and provide reasonable animal models for vaccination studies [6]. LVS, an attenuated strain derived from *F. tularensis* subsp. holarctica (Type B) [7], is the only vaccine against tularemia currently undergoing clinical development in the U.S. [2], [4]. Human challenge studies, as well as use among laboratory workers, suggest that LVS vaccination provides at least partial protection against some forms of the disease, but specific efficacy levels have not been firmly established [2]–[5], [8]–[10]. In contrast, observations suggested minimal protection of people following vaccination with killed *Francisella* despite stimulating production of abundant serum antibodies [3], [8], [11]–[12], similar to experimental studies using mice [13]–[16], especially following aerosol exposure to the most virulent strains.

Examination of broth cultures of all strains of *F. tularensis*, including LVS, on blood agar plates reveals a variety of colony morphologies. These opacity variants suggest bacterial phase variation, a phenotype that may confound evaluation and use of LVS. Lots of LVS that include a high proportion of phase variants have been associated with reduced immunogenicity in humans [4], [17], as suggested earlier in animal studies [7]. Stable opacity variants of LVS, denoted LVS-G and LVS-R, have been isolated *in vitro* [18]. These isolates express alternative chemotypes of *Francisella* LPS, and appear to be analogous to clinical lots with reduced immunogenicity in humans. However, LVS-G and LVS-R have not been tested as vaccines in any experimental models, including mice, to date.

Murine infections with LVS provide a well-established model of infection and immunity against *Francisella*, and indeed for intracellular bacteria generally [19]–[21]. Similar to many intracellular bacteria, LVS infects and replicates primarily in macrophages [22], but exhibits convenient route-dependent virulence in mice [19]. Thus, LVS administered to mice subcutaneously or intradermally (ID) has a high LD_50_ of about 10^6^ and establishes a vaccinating infection, eliciting strong cellular as well as humoral immune responses. However, doses of 10^1^ or more of LVS administered to mice intraperitoneally (IP) or intravenously are lethal. BALB/c or C57BL/6 mice vaccinated ID with 10^4^ LVS survive lethal challenge with LVS of up to 10^6^ IP, and are at least partially protected against parenteral challenge with fully virulent Type A *F. tularensis* SchuS4 [3], [23]–[24]. Also similar to many intracellular pathogens, *in vivo* studies clearly demonstrate that this protection is dependent on T lymphocytes, and involves production of Interferon gamma (IFN-γ), Tumor Necrosis Factor alpha (TNF-α), and nitric oxide (NO). To further uncover T cell effector mechanisms, we have previously developed an *in vitro* tissue culture system to mimic *in vivo* immune responses [25]–[26], in which LVS-immune lymphocytes are co-cultured with LVS-infected bone marrow derived macrophages and intramacrophage bacterial replication is measured. LVS-immune splenocytes, liver leukocytes, and lung leukocytes all control intramacrophage LVS replication *in vitro*, but naive cells do not [27]. In this assay, appropriate T cell subpopulations but not B cells or myeloid cells have activity, and the model appears to faithfully reflect known *in vivo* T cell effector mechanisms [25]–[26].

Here, we take advantage of a panel of *Francisella* vaccine candidates, including LVS, LVS-G, LVS-R, and heat-killed LVS, that induced quantitatively different levels of protection in mice against *Francisella* challenge. These vaccines were chosen to approximate *Francisella* vaccines studied in humans; thus, LVS has been associated with reasonable efficacy, while lots of LVS with higher proportions of opacity variants exhibit reduced immunogenicity (modeled here by the stable variants LVS-G and LVS-R), and killed *Francisella* provided poor protection in humans. Using this panel, we searched for immune responses that predict successful protection. We found that the relative activity of *Francisella*-immune lymphocytes *in vitro* in the co-culture assay, as well as the relative expression of a group of immunologically-related genes in cells recovered from this assay, correlated with the degree of protection observed *in vivo*. This approach to identifying correlates, which couples a functional *in vitro* assay that detects reduction in intracellular bacterial loads with expression of relevant mediators, may be generally applicable to vaccine-induced protection against intracellular pathogens.

## Materials and Methods

### Ethics statement

These studies carried out in strict accordance with the recommendations in the Guide for the Care and Use of Laboratory Animals of the National Institutes of Health. All experiments performed using LVS only were conducted under protocols approved by the Animal Care and Use Committee (ACUC) of CBER. Experiments including *F. tularensis* SchuS4 challenge were performed at the Rocky Mountain Laboratories (RML) under protocols approved by the RML ACUC. Both sets of protocols stressed practices and procedures designed to strictly minimize any suffering.

### Experimental animals

Six to twelve week old wild type male C57BL/6J mice were purchased from Jackson Laboratories (Bar Harbor, ME). All mice were housed in sterile microisolator cages in a barrier environment at CBER/FDA, fed autoclaved food and water *ad libitum*, and routinely tested for common murine pathogens by a diagnostic service provided by the Division of Veterinary Services, CBER. Within an experiment, all mice were age matched.

### Bacteria and growth conditions

*F*. *tularensis* strain SchuS4, provided by Jeannine Peterson, (Centers for Disease Control, Fort Collins, CO); *F. tularensis* LVS (American Type Culture Collection 29684); *F. tularensis* LVS-G and LVS-R, originally obtained from Francis Nano (University of Victoria, Victoria, British Columbia, CA); and *Listeria monocytogenes* strain EGD (ATCC 15313) were all grown to mid-log phase in modified Mueller-Hinton (MH) broth (Difco Laboratories, Detroit, MI), as previously described [28], harvested, and frozen in 1 ml aliquots in broth alone at −70°C. For each bacterial stock, separate experiments determined numbers of live colony forming units (CFU), confirmed typical colony morphologies, and confirmed expected LD_50_s and times to death using adult male BALB/cByJ mice (which are the most sensitive strain for quality control testing). Bacteria were periodically thawed for use, and viability was quantified by plating serial dilution on modified MH agar plates. Aliquots of *F. tularensis* LVS were heat killed at 56°C for 30 minutes immediately prior to use, and complete killing confirmed by plate count.

### Bacterial immunizations and challenge

Groups of mice were immunized by intradermal (ID) injection with 1×10^4^ CFU LVS, 1×10^4^ LVS-G, 1×10^4^ LVS-R, or an amount equivalent to 1×10^8^ heat-killed LVS; doses of each were optimized in initial experiments for maximal protection against lethal IP LVS challenge. All vaccines were diluted in 0.1 ml phosphate-buffered saline (PBS) (BioWhittaker, Walkersville, MD) containing <0.01 ng of endotoxin/ml. Actual doses of inoculated bacteria were retrospectively determined by plate count; control groups received 0.1 ml PBS ID. As indicated, four – twelve weeks after vaccination, mice were challenged with 10^3^ – 10^6^ LVS intraperitoneally (IP), or 50 CFU SchuS4 subcutaneously (SC), and monitored for survival. Animals were euthanized when clearly moribund.

### Preparation of splenocytes

Single-cell suspensions of splenocytes were generated for *in vitro* culture, flow cytometry, and qRT-PCR analysis by standard techniques, and had no detectable CFU at the time of harvest. Viability was assessed by exclusion of trypan blue and flow cytometry (see below).

### Infection of bone marrow–derived macrophages with *F. tularensis* LVS and co-culture with splenocytes

Co-cultures were performed in 24 well tissue culture plates as described previously [25]–[27], [29]–[31]. Briefly, bone marrow macrophages (BMMØ) were cultured in complete DMEM (DMEM supplemented with 10% heat-inactivated FCS [HyClone, Logan, UT], 10% L-929-conditioned medium, 0.2 mM L-glutamine, 10 mM HEPES buffer, and 0.1 mM nonessential amino acids) in 24 well plates. Confluent adherent macrophage monolayers were infected for 2 hours with *F. tularensis* LVS at a multiplicity of infection (MOI) of 1∶20 (bacterium-to-BMMØ), washed, treated for 60 min with 50 µg/ml gentamicin, and washed extensively with antibiotic-free medium. Single-cell suspensions of splenic lymphocytes derived from vaccinated mice (5×10^6^/well, or as indicated) were added to confluent LVS-infected macrophages (∼1×10^7^/well) [25]–[26]. At the indicated time points, non-adherent cells were harvested, centrifuged and assessed for changes in cell surface phenotype by flow cytometry or gene expression by qRT-PCR as described below. Supernatants from harvested cells was collected and stored at −70°C until analyzed for nitric oxide and cytokines as described below. Intracellular bacterial loads in adherent macrophages were determined as previously described. Additional macrophages were collected following incubation in 0.05% trypsin/EDTA for 5 minutes, followed by neutralization with complete media containing serum.

### Real time PCR

Cells to be assessed for changes in gene expression by qRT-PCR were pelleted by centrifugation for 10 minutes at 1000 rpm, immediately immersed in RNA*later* (Ambion, Austin, TX) and stored at −70°C until further characterization. Total RNA was extracted from samples using RNeasy mini kits (Qiagen, Valencia, CA), according to the manufacturer's directions. RNA quality and concentration were assessed by Bioanalyzer, including calculation of the RNA Integrity Number (RIN) via a software algorithm that estimates RNA sample integrity from elements in the bioanalyzer electrophoretic trace, and then assigns a score to RNA quality between 0 and 10 (Agilent Technologies, Santa Clara, CA). One microgram of RNA was used to synthesize cDNA using the commercially available kit RetroScript Reverse Transcription for RT-PCR (Ambion, Applied Biosystems Foster City, CA), following the manufacturer's instructions. Semi-quantitative real-time PCR amplification was completed with an ABI Prism 7000 sequence detection system (Applied Biosystems, Carlsbad, CA). For initial screening of genes' expression, cDNA prepared from non-adherent cells was diluted and used to amplify a panel of genes of immunological interest (e.g., Th1-Th2-Th3 RT^2^ Profiler PCR Array System, SABiosciences, Frederick, MD), following the manufacturer's instructions. To validate the initial array qRT-PCR results, a second series of independent amplifications for selected genes were performed. Independent primers and probes were purchased from Applied Biosystems. cDNA was initially diluted to 100 µl and then two µl of each cDNA was further diluted to a volume of 25 µl PCR mix (Applied Biosystems) containing 0.1 µM and 0.2 µM of each primer and probes, respectively. Serial dilutions of each individual gene were used to generate a Glyceraldehyde phosphate dehydrogenase (GAPDH) standard curve. For PCR amplifications, the initial denaturation at 95°C for 10 minutes were followed by 40 cycles of 95°C for 15 seconds and 60°C for 1 minute. The level of mRNA of each gene relative to the GAPDH mRNA concentrations was calculated by plotting the crossing point (Ct) of each amplification in relationship to the GAPDH standard curve. Delta Ct (ΔCt), and the ratio between ΔCt of vaccines' samples and control samples (ΔΔCt), were then calculated.

### Flow cytometry

Single cell suspensions prepared from spleens and splenocytes recovered from co-culture after the indicated time of culture were stained for a panel of murine cell surface markers and subjected to multiparameter analyses using a Becton-Dickinson LSR II flow cytometer (San Jose, CA) and FlowJo (Tree Star, Inc) software essentially as previously described [29]–[30]. Briefly, cells were washed and resuspended in flow cytometry buffer (PBS/2% serum), and non-specific binding of antibodies was inhibited by blocking Fc receptors with anti-CD16 (Fc Block; BD Pharmingen). To discriminate live from dead cells, a staining step for dead cells was performed using a commercially available kit and following manufacturers' instruction (Live/Dead Staining Kit, Invitrogen). The cells were then washed and stained for cell surface markers. Antibody concentrations were optimized separately for use in seven- to nine-color staining protocols as required, using appropriate fluorochrome-labeled isotype matched control antibodies. The following antibodies were used: anti-B220 (clone RA3-6B2), anti-CD19 (clone 1D3), anti-TCRβ (clone H57-597), anti-CD4 (clone RM4-5), anti-CD8β (H35-17.2), anti-NK1.1 (clone PK136), anti-CD11b (clone M1/70), anti-Gr-1 (clone RB6-8C5), and anti-CD11c (cloneHL3), each labeled with a variety of fluorochromes as needed (above antibodies were purchased from BD Pharmingen).

### Statistical analyses

Kaplan Meier curves were plotted to compare time to death following lethal LVS challenge between different vaccine groups, and log-rank (Mantel-Cox) analyses calculated to compare survival of different groups (using Prism, GraphPad Software, La Jolla, CA). Linear regression models were fit to compare the effects of splenocytes on controlling bacterial growth from different vaccine groups while adjusting for splenocyte concentration. Univariate and multivariate logistic regressions were used to correlate protection against lethal LVS challenge with either fold change in gene expression at two different time points (∼6 weeks after vaccination and ∼12 weeks after vaccination), or with all data combined across both time point using standardized scores of gene expression. The results of these analyses were quite similar, and thus the results using all data combined are shown here. Standardized scores were used to protect against the possibility that the relative magnitude of gene expression for any given gene might be relatively different at the early time point compared to the late time point. Standardized scores were obtained by subtracting the average log expression level and then dividing by the standard deviation of the log expression level in the same experiment. The Akaike information Criterion was used to compare different logistic regression models. Pearson's correlation coefficients of standardized scores of expression level for pairs of genes were reported. All p-values were two-sided, and p-values<0.05 were considered to be statistically significant. Data analyses were conducted using R (R Foundation for Statistical Computing, Vienna, Austria).

### Optimization of conditions for analyses

Initial studies optimized conditions for these experiments; further characterization and modifications of previously published *in vitro* co-culture methodologies [25]–[26] were required to be most appropriate for the present purposes. Detailed information regarding data supporting the resulting modifications is provided in Supporting Information (see Supporting Information, Text S1, Figures S1–S3, and Table S1).

## Results

### Determination of the ability of *in vitro* co-cultures to reflect the hierarchy of protection stimulated by qualitatively different *Francisella* vaccines

**Figure 1:**
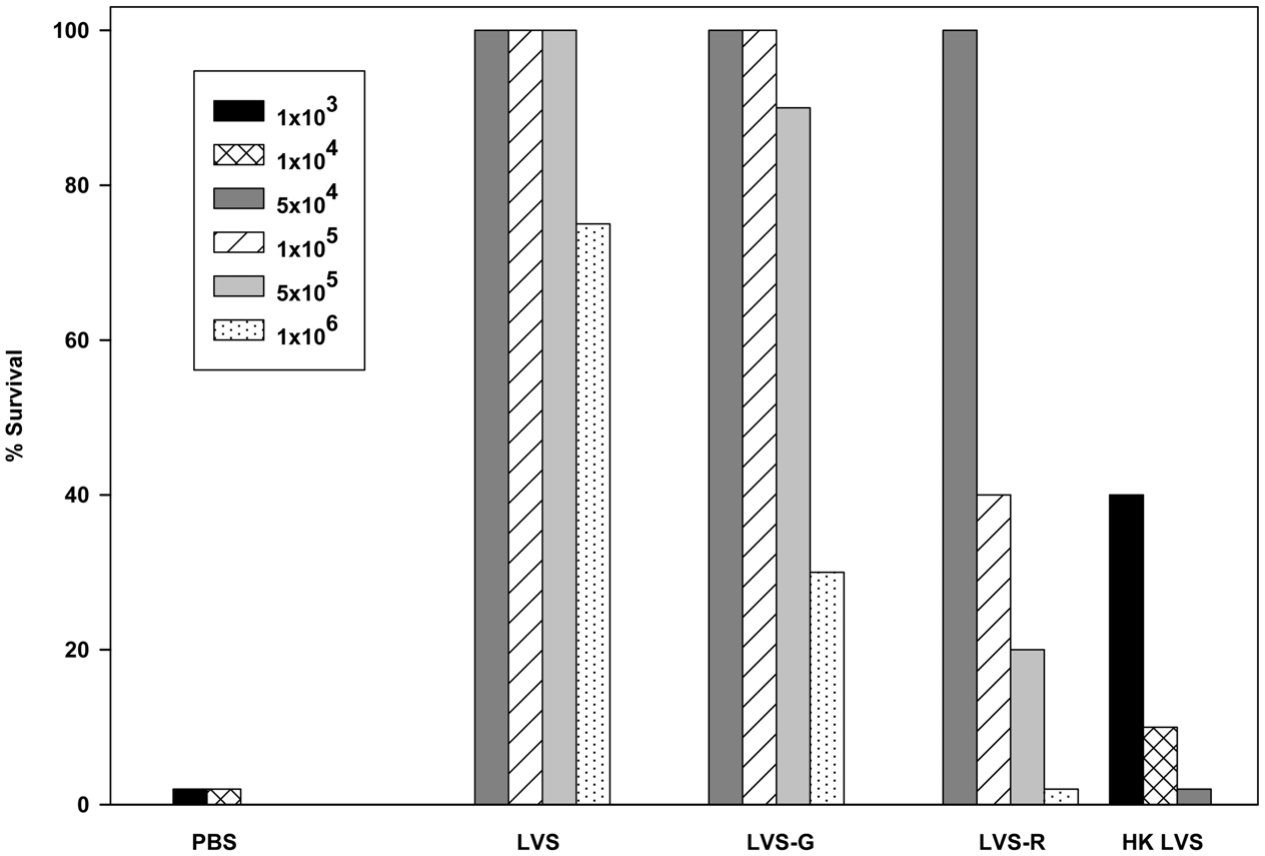
Hierarchy of strength of protection against lethal systemic *F. tularensis* LVS challenge after vaccination of mice with LVS or the variants LVS-G, LVS-R, and heat-killed LVS. C57BL/6J mice were immunized by ID infection with 1×10^4^ CFU LVS, 1×10^8^ HK-LVS, 1×10^4^ LVS-G, or 1×10^4^ LVS-R. At 6 weeks after vaccination, mice vaccinated with PBS were challenged with 10^3^ or 10^4^ LVS IP; mice vaccinated with HK-LVS were challenged with 10^3^, 10^4^, or10^5^ LVS IP; and mice vaccinated with LVS, LVS-G, and LVS-R were challenged with 5×10^4^, 10^5^, 5×10^5^, or 10^6^ LVS IP, as indicated for each group, and monitored for survival. Results shown are from one representative experiment of three independent experiments of similar design with similar outcome.

To determine whether the *in vitro* co-culture system may serve as a functional correlate of protection, we first identified a panel of vaccine candidates that provided different degrees of protection against lethal *Francisella* challenge *in vivo*. C57BL/6J mice were vaccinated ID with 10^4^ LVS, with 10^4^ of the opacity variants LVS-G or LVS-R, or with 10^8^ -heat killed (HK-) LVS, and then challenged one month after vaccination with increasing lethal doses of LVS IP (Figure 1). All mice vaccinated with wild type LVS survived challenge with up to 5×10^5^ LVS IP, and 75% survived challenge with the highest dose tested, 10^6^ LVS IP. In contrast, mice vaccinated with LVS-G exhibited 90% survival following challenge with 5×10^5^ CFU and 30% following challenge with 1×10^6^ CFU. Mice vaccinated with LVS-R exhibited only 20% survival following challenge with 5×10^5^ CFU, and none survived challenge with 10^6^ CFU. Finally, vaccination with HK-LVS failed to protect against challenge with 5×10^4^ CFU or higher. In later experiments, a single challenge dose of 5×10^5^ - 10^6^ LVS IP was chosen as appropriate for discriminating between the degree of protection provided by all vaccines in the panel.

**Figure 2:**
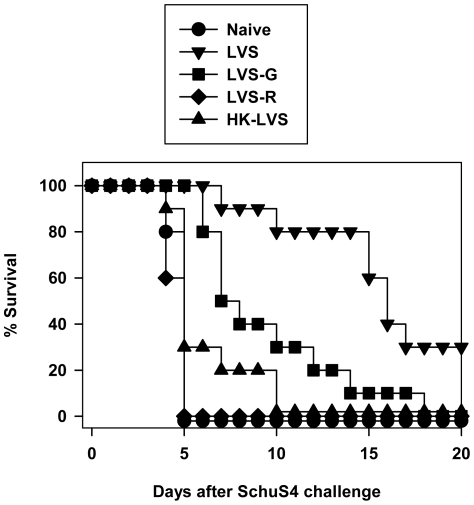
Hierarchy of survival against lethal systemic *F. tularensis* SchuS4 challenge after vaccination of mice with LVS or the variants LVS-G, LVS-R, and heat-killed LVS. Groups of five C57BL/6J mice were immunized by ID infection with 1×10^4^ CFU LVS, 1×10^4^ LVS-G, 1×10^4^ LVS-R, or 1×10^8^ HK-LVS. At 4 weeks after vaccination, all mice were challenged with 50 CFU SchuS4 subcutaneously (SC), and monitored for survival; all mice that survived through day 20 were long term survivors. The combined results of two independent experiments, using 10 total mice per vaccine group, are shown. By log-rank (Mantel-Cox) analysis, vaccination with LVS and LVS-G significantly improved survival (both p<0.0001 compared to naive mice), and were significantly different from each other (p = 0.0041); vaccination with HK-LVS or LVS-R did not significantly improved survival compared to naive mice (p = 0.0968 or p = 0.3425, respectively), but both of these were significantly different compared to vaccination with LVS or LVS-G (p<0.0001 for all combinations).

These data indicated that this panel of candidate vaccines exhibited a hierarchy of relative protection against *in vivo* lethal LVS challenge, such that LVS>LVS-G>LVS-R>HK-LVS>PBS (naive control). We next examined the protective capacity of these vaccines against lethal parenteral challenge with a selected dose of fully virulent Type A *F. tularensis* (SchuS4). Similar to the outcome using LVS challenge, and consistent with previous reports [32], approximately 30% of mice vaccinated with LVS survived this dose of SchuS4 challenge and time to death of those that died was greatly extended, while vaccination with LVS-G protected only 10% of mice against lethal SchuS4 challenge (Figure 2). In contrast to challenge with LVS, vaccination with LVS-R did not provide detectable protection against this dose of SchuS4 challenge, and vaccination with HK-LVS only slightly extended time to death. Nonetheless, for this group of vaccines, a similar hierarchy of protective efficacy found following challenge with wild type LVS was also found using challenge with fully virulent *Francisella*.

**Figure 3:**
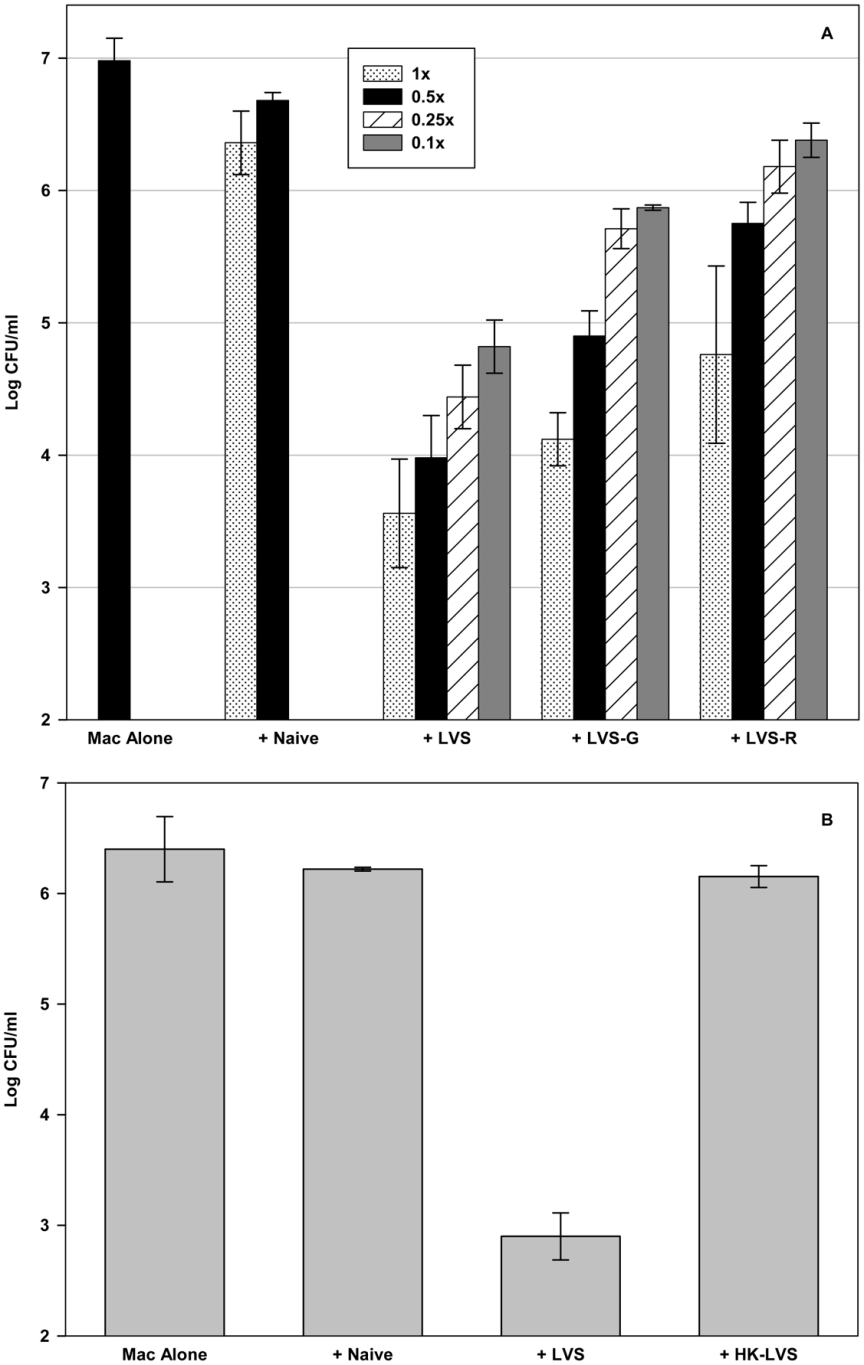
Splenocytes from mice vaccinated with a panel of LVS-related vaccines exhibit a hierarchy of control of intramacrophage LVS growth. (A) BMMØs from C57BL/6J mice were infected with LVS at an MOI of 1∶20 (bacterium-to-macrophage ratio; “Mac alone”), and co-cultured with the indicated numbers of splenocytes obtained from either naive C57BL/6J mice or C57BL/6J mice vaccinated 1×10^4^ CFU LVS, 1×10^4^ LVS-G, or 1×10^4^ LVS-R 6 weeks previously. Here, for all co-cultures containing added splenocytes, “1x” = 5×10^6^ splenocytes per well (used in all previous experiments), and 0.5x, 0.25x, and 0.1x refer to corresponding decreases in the total number of added splenocytes. (B) BMMØs from C57BL/6J mice were infected with LVS at an MOI of 1∶20 (bacterium-to-macrophage ratio), and co-cultured with splenocytes obtained from either naive C57BL/6J mice or C57BL/6J mice vaccinated 1×10^4^ CFU LVS or 1×10^8^ HK-LVS 6 weeks previously. For both A and B, after three days of co-culture, BMMØ were washed, lysed, and plated to determine the recovery of intracellular bacteria. Values shown are the mean numbers of CFU/ml ± SD of viable bacteria for triplicate samples. Results shown are from one representative experiment of three (A) or four (B) independent experiments of similar design with similar outcome.

In parallel with *in vivo* vaccination and challenge studies, the activities of splenocytes obtained from mice vaccinated with LVS, LVS-G, LVS-R, HK-LVS, or PBS (control) were compared. To determine the relative effectiveness of each type of primed cells, decreasing numbers of splenocytes were added to a constant number of LVS-infected macrophages. As seen in Figure 3A, on a per-cell basis, cells obtained from LVS-infected mice were most effective in controlling the intramacrophage growth of LVS; those from LVS-G vaccinated mice were less effective, and those from LVS-R-vaccinated mice the least effective. The relationship between relative control by cells from the different groups was then investigated. A linear regression with indicators of different vaccine groups and the cell concentration as covariates was used to compare log CFU of recovered bacteria in different vaccine groups, adjusting for the levels of cell concentrations (Figure S4). The result of regression analysis demonstrated that, for any fixed cell concentration, cells from LVS-G-vaccinated mice were significantly less effective in controlling bacteria growth, a difference of about 0.95 log, compared to those from LVS-vaccinated mice (p<0.001). Similarly, cells from LVS-R-vaccinated mice were about 1.57 log less effective than cells from LVS-vaccinated mice (p<0.001). Finally, cells from LVS-R-vaccinated mice were 0.62 logs less effective than from those from LVS-G-vaccinated mice (p<0.001). As seen previously [25]–[27], [29]–[31], the results suggested that co-cultures containing cells from naive mice exhibited minimal and inconsistent reductions in bacterial numbers compared to those with LVS-infected macrophages alone (e.g., Figure 3A, p = 0.02; Figure 3B, p = 0.35); further, there was no significant relationship between the concentration of naive cells and bacterial numbers. Finally, cells obtained from mice vaccinated with HK-LVS did not significantly inhibit bacterial growth control compared to either naive cells or cultures with LVS-infected macrophages only, even at the highest cell numbers tested (Figure 3B). Thus, the hierarchy of *in vitro* activities of cells from vaccinated mice was again LVS>LVS-G>LVS-R>HK-LVS. Further, because only LVS-immune T cells are active in this setting, these data estimate the relative frequencies of memory T cells.

Supernatants and cells were also recovered on day 2 from each type of co-culture. Supernatants were then analyzed as above for cytokine production by ELISA and NO production by Griess reaction; cells were characterized by flow cytometry; and mRNA prepared from recovered cells was analyzed for relative gene expression. The amounts of TNF-α, IFN-γ, and NO produced were consistent with previously published studies using LVS vaccination alone [25]–[27], [29]–[31]. Here, relative cytokine and NO production exhibited the same pattern as that observed in the survival studies and in *in vitro* control of intramacrophage LVS replication, such that LVS>LVS-G>LVS-R>HK-LVS and naive groups (data not shown). Flow cytometry analyses of recovered cells confirmed the relative enrichment of T cells (similar to that illustrated in Table S1), and did not reveal any obvious differences between vaccine groups (data not shown). Collectively, these data indicated that the hierarchy of protective capacity engendered by *in vivo* vaccination with this panel of vaccines was faithfully reflected by the relative ability of each type of *Francisella*-immune splenocytes to persist in culture, produce relevant cytokines and nitric oxide, and ultimately to effect control of intramacrophage bacterial growth.

### Identification of genes whose relative expression following *in vitro* co-culture reflects the hierarchy of protection stimulated by qualitatively different *Francisella* vaccines

Because these *in vitro* co-culture conditions reliably detected differences in vaccine quality, non-adherent immune splenocytes from all groups were recovered on day two from each co-culture, and then analyzed in detail for relative gene expression. For these experiments, groups of mice were vaccinated with LVS, LVS-G, LVS-R and HK-LVS. At either 6 weeks or 12 weeks after vaccination, some mice were challenged *in vivo*, and other mice were sacrificed at the same time to prepare splenocytes, perform *in vitro* co-cultures, and recover non-adherent cells for mRNA analyses. Similar to initial studies using cells from naive or LVS-vaccinated mice only (see Supporting Information text), the relative mRNA expression of genes of immunologic interest in splenocytes from mice vaccinated with LVS, LVS-G, LVS-R, or HK-LVS was compared to that of splenocytes from naive mice by RT-PCR, using commercially available arrays that included immunologically-related genes (e.g., Profiler PCR Th1-Th2-Th3 array). Data generated from initial experiments using the complete vaccine panel revealed that some genes, such as GF1 and CCR4, which in initial studies were up-regulated in LVS-primed cells compared to naive cells, were either inconsistent or up-regulated to a similar degree for all vaccines and did not exhibit a differential pattern (Table S2, “SYBR”). In contrast, other genes appeared to exhibit a range of expression that reflected the relative effectiveness of vaccination. For example, IFN-γ appeared to be highly expressed in LVS-primed cells as well as in cells from LVS-G-vaccinated mice, only moderately expressed in cells from LVS-R-vaccinated mice, and expressed very little in cells from HK-LVS-vaccinated (all compared to naive mice). Similar to IFN-γ, the relative expression of several other genes, such as IL-6, TNF-α, IL-18bp, and GM-CSF, reflected the relative level of both *in vivo* protection and *in vitro* bacterial growth control activities of the different vaccines.

**Figure 4:**
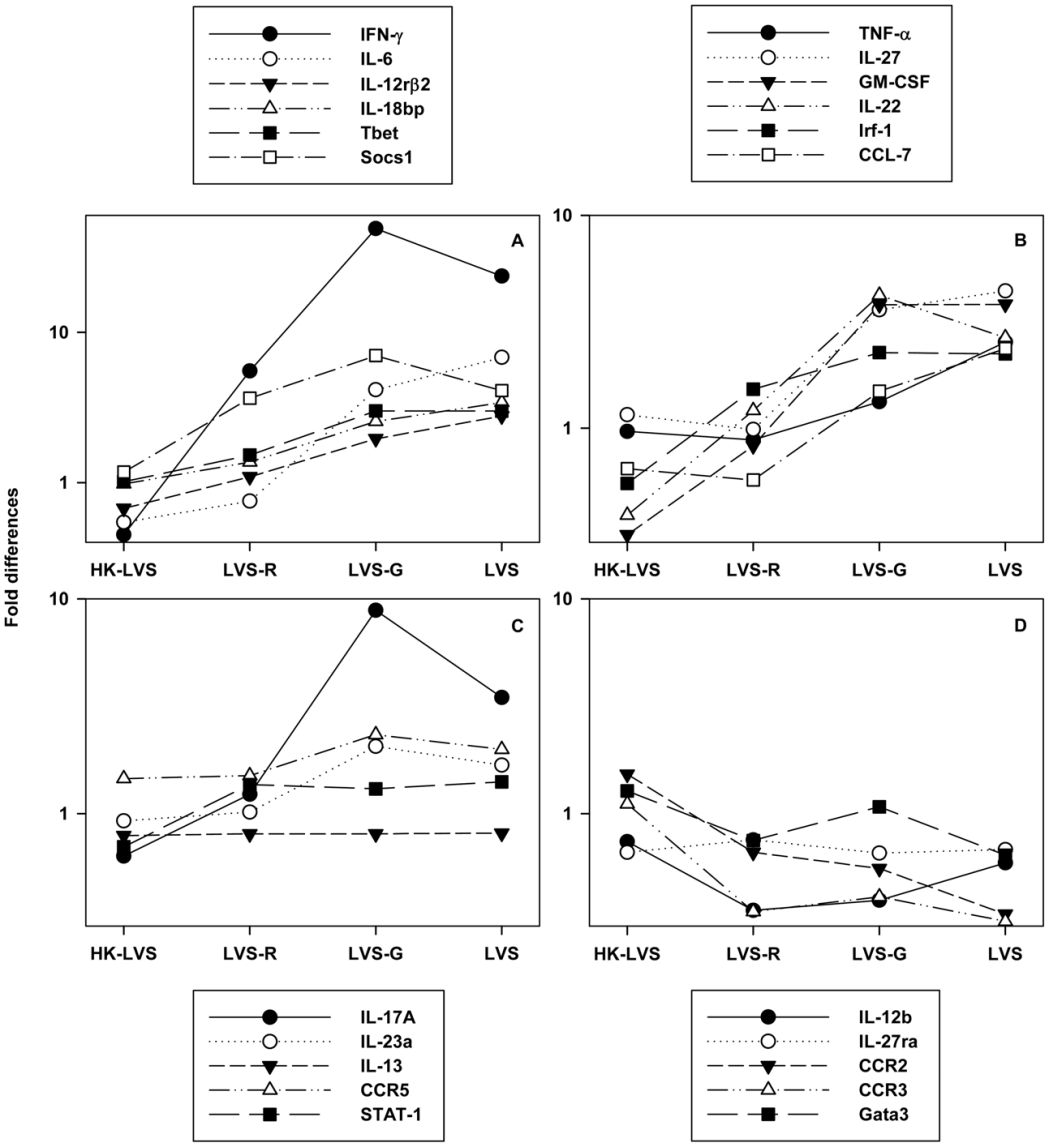
Relationship between relative differences in gene expression and type of vaccine for differentially regulated genes. Data from all four experiments using the full panel of vaccine candidates (Table S2, experiments 4 – 7) are used to provide a graphic representation of the relationship between type of vaccine and relative gene expression. All data were normalized in relationship to endogenous GAPDH standard curves, and values shown are median of the fold differences (ΔΔCt) in relationship to naive cells for each type of vaccine, as indicated. Panel A depicts the genes designated as Group 1 in Table 1 (all significant, p<0.0001); Panel B depicts the genes designated as Group 2 in Table 1 (all significant, p<0.0001); Panel C depicts the remaining up-regulated genes not included in Groups 1 or 2 of Table 1 (IL-17A, IL-23a, CCR5, and STAT-1 significant with p<0.05, but IL-13 not significant; see Table S4 for exact p values); and Panel D depicts down-regulated genes (all non-significant; see Table S4 for p values). The corresponding legends are shown immediately above or immediately below each panel.

The initial comparisons focused attention on a group of 15 genes with apparently differential expression patterns, either in terms of relative up-regulation or relative down-regulation. Seven other genes were also considered of ongoing interest, either because they were not included on the commercial panel used and had a known biological relationship to other genes that were correlated with protection (e.g., IL-22), and/or because results were ambiguous (e.g., IL-17A and IL-13). A set of 22 genes were therefore selected for more detailed studies, and separate primer-probe sets prepared to perform qRT-PCR analyses. This approach was applied to again analyze mRNA from previous experiments, as well as mRNA from additional independent experiments. Collectively, these included two independent experiments using splenocytes from mice vaccinated six weeks earlier (Table S2, experiments 4 and 6) and two independent experiments using mice vaccinated twelve weeks earlier (Table S2, experiments 5 and 7). For analysis of qRT-PCR data from selected genes, Ct results were normalized to a standard curve of GAPDH before calculation of ΔCt and fold changes (ΔΔCt) between samples from naive and vaccinated mice (ΔΔCt). When compared for up-regulated genes, the fold change for most of these genes in cells from LVS-vaccinated mice was similar to those in cells from LVS-G-vaccinated mice; both were greater than those in cells from LVS-R vaccinated mice, which in turn were greater than those in cells from HK-LVS vaccinated mice or naive mice (Figure 4, panels A–C). In contrast, when the relative fold change (ΔΔCt) of putatively down-regulated genes was compared across different experiments, the amounts of differences were relatively modest (Figure 4, panel D), similar to observations made using Profiler PCR arrays. Taken together, using a different detection system (including probes instead of SYBR Green) and a different normalization approach, we again found clear relationships between the relative expression levels of most members of this panel of up-regulated genes and the hierarchy of vaccine protection. Further, the latter method allowed more accurate quantification of relative gene expression.

### Specificity of relative gene expression following *in vitro* co-culture

To examine whether gene expression patterns observed are specific for *F. tularensis* LVS vaccination and related to *Francisella* vaccine efficacy, C57BL/6J mice were vaccinated ID with LVS, LVS-R, HK-LVS, or 5×10^3^ *Listeria monocytogenes*. Two weeks later, mice were either challenged with lethal dose of 10^6^ LVS IP, or used to prepare spleen cells that were co-cultured with LVS-infected macrophages, recovered after two days, and analyzed for relative gene expression. For the LVS related vaccines, the same patterns of survival and *in vitro* growth control were found (data not shown). In contrast, *Listeria*-vaccinated splenocytes did not significantly reduce the intramacrophage growth of LVS compared to control co-cultures (data not shown; and see [25]), and *Listeria*-vaccinated splenocytes exhibited an absence of up- or down-regulation. The fold change (ΔΔCt) values using *Listeria*-vaccinated splenocytes were mostly similar to those observed in cells from mice vaccinated with HK-LVS. For example, IFN-γ up-regulation in cells from LVS-vaccinated mice was about 22 fold compared to naive mice; 7.2 in cells from LVS-R-vaccinated mice; 0.8 in cells from HK-LVS vaccinated mice; and 1.7 in cells from *Listeria*-vaccinated mice. Thus, the working panel of genes specifically reflected activities of *Francisella*-immune cells.

### Integrated statistical analyses of the ability of relative gene expression following *in vitro* co-culture to reflect the hierarchy of protection stimulated by qualitatively different vaccines

**Figure 5:**
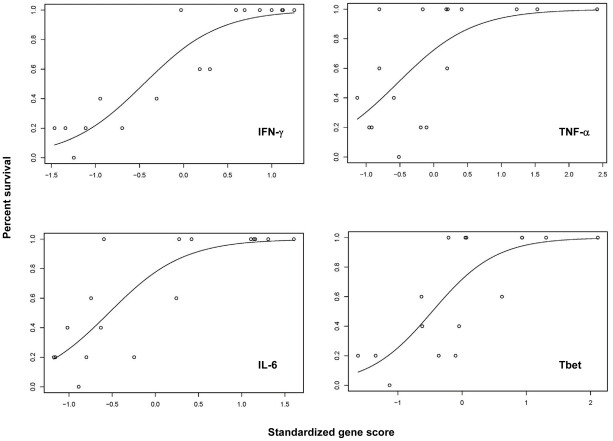
Relationship between gene expression and survival across all vaccines studied at six weeks after vaccination for IFN-γ, TNF-α, IL-6, and T-bet. Data from Table S2, all experiments, were analyzed by logistic regression across all vaccines and all time points as described in the text. For each individual gene, the plotted results shown depict the percent survival of LVS-challenged mice as a function of the standardized score of the log_10_ fold difference in gene expression. All mediators depicted here exhibited a significant positive relationship between the degree of gene expression and survival (see Table 1 and Table S4).

The resulting data derived from both the Profiler arrays and from the selected genes assessed by qRT-PCR were used to examine statistical correlations between survival and *in vitro* gene expression in response to the different vaccines (see Table S2, all experiments). For each gene, the proportion of surviving mice was plotted against a standardized score of gene expression. Examples of these relationships are illustrated in Figure 5, in which analyses of IFN-γ, TNF-α, IL-6, and T-bet relative mRNA expression by qRT-PCR are shown; p values for the relationship between survival and all genes analyzed by Profiler array or qRT-PCR are provided as Supporting Information (Tables S3 and S4, respectively). Of the 22 selected genes, 20 were significantly related to survival according to Profiler array data, validating the initial selection, but only 16, were significantly related to survival using qRT-PCR data.

The logistic regressions analyses using data from the Profiler arrays (Table S2, SYBR) indicated that about 20 other genes, in addition to the 22 genes already selected, were also significantly related to survival. These included genes that were up-regulated (e.g., SOCS3 and Tmed) and some that were down-regulated (e.g., Jak1 and CD27). However, the fold change of each of these genes even for LVS-immune cells compared to naive cells was relatively small, <2 for up-regulated genes and >0.5 for down-regulated genes. Moreover, the range of differences for these genes across all vaccines was judged too small to be reliably useful in this context, and these candidates have not yet been pursued further.

Taken together, the genes that exhibited consistent changes of useful magnitude across all experiments, as well as significant pairwise correlations of the relative degree of expression between all possible pairs of genes (Table S5), included IFN-γ, IL-6, IL-12rβ2, T-bet (Tbx21), Socs1, and IL18bp (Table 1, Group 1; Figure 4, panel A). Genes with notable, but less universal, associations were GM-CSF, IL-27, TNF-α, IL-27, and Irf1 (Table 1, Group 2; Figure 4, panel B; remaining up-regulated genes are illustrated in Figure 4, panel C, and down-regulated genes in Figure 4, panel D).

**Table 1:** Working panel of proposed correlates of protection for the intracellular bacterium *Francisella.*

Mediator	Fold change (range) compared to naive*			
	LVS	LVS-G	LVS-R	HK-LVS
**Group 1:**				
IFN-γ	23.8 (13 – 359)	49 (12 – 347)	5.5 (0.8 – 33)	0.5 (0.2 – 1.2)
IL-6	6.8 (2.5 – 8)	4.1 (0.8 – 7)	0.8 (0.5 – 2)	0.5 (0.4 – 1.2)
IL-12rβ2	2.8 (1.6 – 4)	2.0 (1.2 – 5)	1.1 (0.7 – 1.8)	0.7 (0.5 – 1.1)
T-bet	3.0 (1.7 – 10)	3.0 (1.9 – 5)	1.5 (0.9 – 3)	1.0 (0.4 – 2)
IL-18bp	3.4 (3.1 – 7)	2.6 (0.7 – 6)	1.4 (1.3 – 1.7)	1.0 (0.4 – 1.7)
SOCS-1	4.1 (2.7 – 15)	7.0 (3 – 17)	3.6 (1.0 – 5)	1.2 (0.8 – 1.6)
**Group 2:**				
GM-CSF	3.8 (0.4 – 10)	3.8 (1.5 – 12)	0.8 (0.4 – 2.3)	0.3 (0.3 – 1.7)
TNF-α	2.5 (1.4 – 3)	1.3 (0.8 – 4)	0.9 (0.7 – 1.8)	1.0 (0.8 – 1.1)
Irf-1	2.2 (1.5 – 4)	2.3 (1.3 – 5)	1.5 (1.8 – 3)	0.5 (0.5 – 1.64
IL-22	2.7 (0.4 – 14)	4.2 (1.1 – 13)	1.2 (0.4 – 3)	0.4 (0.1 – 1.2)
IL-27	4.4 (2.2 – 7)	3.3 (0.7 – 8)	1.0 (0.9 – 3)	1.1 (0.5 – 1.7)
CCL7 (MCP-3)	2.4 (1.9 – 2.6)	1.5 (0.3 – 1.9)	0.6 (0.5 – 0.7)	0.6 (0.3 – 2.0)

*The median fold change of the indicated mediator for each indicated vaccine group is shown, with the range across all experiments in parenthesis. For the range in parentheses, fold change values over 2 were rounded to the nearest whole number, and those less than 2 were rounded to one decimal place. Results from 4 total experiments were included (see Table S2). p values for logistic regressions of associations between degree of gene expression and degree of survival were all<0.001 (see Table S4).

Finally, to assess whether fold changes in mRNA expression for different genes would work in concert and better predict survival than single genes, multivariate logistic regression analyses were performed. Although specific pairs of genes displayed correlative degrees of expression, multivariate analyses of gene pairs (Table S6) did not provide stronger associations with survival than any one gene alone, based on the Akaike information criterion value. Likewise, groups of three genes did not provide any statistically significant improvement in survival prediction than did the models with one or two genes (data not shown).

## Discussion

Currently there are no validated options for predicting protection against intracellular pathogens. Human clinical field trials for many intracellular pathogens will be difficult, either because of the long time to develop disease (i.e., tuberculosis), or the sporadic nature of disease in nature (i.e., tularemia). A recent FDA regulation provides an option for evaluating vaccine efficacy using animal studies under special, well-defined circumstances that may most likely be applicable to biodefense pathogens [33]; but a rational means of bridging efficacy between animals and humans and extrapolating vaccine dose will be critical. Many such issues could potentially be addressed by derivation of correlates of protection that can be measured in several species. The word “correlate” has been ascribed a wide variety of definitions [1], [34]; here, we use the term to refer to a measurement that detects relevant biological functions critical for, and statistically related, to protection against an infectious disease [35].

Historically, efforts to identify and measure relevant serum antibodies have failed to successfully predict vaccine-induced protection, particularly for replicating, live attenuated vaccine. Another approach is to relate the quantity of an immune parameter to the degree of protection. Production of IFN-γ *ex vivo* has been extensively explored, particularly in studies of *M. tuberculosis*. However, there are many human clinical and experimental examples where the relative levels of IFN-γ measured do not reflect the degree of successful vaccination [36]–[41]. The collective evidence instead indicates that it is likely that local availability of IFN-γ is necessary, but not sufficient, for protection. More recently, “multi-functional T cells” that exhibit the ability to simultaneously produce IFN-γ, TNF-α, and IL-2, have been proposed as vaccine correlates [42]–[43]. While promising in the context of mouse models of *Leishmania* infections [44] and some studies of tuberculosis vaccines [45]–[47], in other cases no obvious correlation has been detected between MFCs and protection [48]–[51]. Efforts to develop genomic and metabolomic biomarker signatures for tuberculosis infection and vaccination, particularly in the context of HIV/AIDS, are underway [52]. Although still complex, the approach illustrated here of coupling a panel of qualitatively different vaccines, combined with *in vitro* re-stimulation via co-cultures and semi-quantitative mRNA analyses, clearly identified mediators that correlate strongly with the relative degree of vaccine-induced protection against lethal challenge *in vivo*.

The *Francisella* infection and immunity model offered the advantage of having a panel of different vaccine candidates, coupled with the ability to more precisely define the strength of protection *in vivo* by using a range of lethal challenge doses. Vaccination of humans with LVS engenders production of *Francisella*-specific serum antibodies, as well as memory T cells in peripheral blood that produce IFN-γ, IL-17A, and IL-22 following antigen stimulation [53]–[56], but these are currently of unknown contribution to protection of people. To date, there are limited studies regarding correlates of immunity to *Francisella*. Vaccination mice with static *Francisella* vaccine candidates, including outer membrane protein preparations or ethanol-inactivated LVS formulated with Freund's adjuvant, provided partial protection against respiratory challenge with 40 CFU of fully virulent type A *F. tularensis*, accompanied by production of serum antibodies and large levels of TNF-α and IL-2 in sera of vaccinated mice after challenge [57]. In studies using a mouse model to compare intradermal vaccination of mice with LVS to vaccination with genetically attenuated mutants of SchuS4, protection against challenge with fully virulent *F. tularensis* was not correlated with levels of serum IFN-γ or IgM/IgG antibodies [58]; only pulmonary IL-17 quantities after secondary challenge appeared to track with protection [59]. Here, LVS-G and LVS-R, spontaneous variants that express alternate LPS chemotypes, proved to provide intermediate levels of *in vivo* protection (Figure 1). Because antibodies to LPS likely play a minor role in protection against lethal *Francisella* challenge even in mice [60]–[61], the reduced protection is unlikely to be explained completely by reduced serological responses. More likely, reduced protection is explained either by reduced persistence and total antigen exposure in these serum-sensitive variants [18]; or, changes in LPS expression are a visible marker for simultaneous changes in expression of other bacterial genes that are important in protection. Of note, it is likely the mechanisms of protection provided by the live attenuated strains LVS, LVS-G, and LVS-R are similar, but the strengths of protection quantitatively different; we consider this an important feature that is critical to permitting strong interpretations across different vaccines.

Using this panel, a hierarchy of strength of protection was evident using *Francisella* LVS challenge of mice (Figure 1). Although it was not feasible to perform larger experiments using graded doses of challenge with fully virulent *F. tularensis* SchuS4, the proportion of vaccinated survivors and differences in times to death following challenge with one selected dose supported a similar hierarchy (Figure 2). Tangentially, these comparisons also suggest that lethal parenteral challenge of vaccinated mice with LVS could serve as an informative screen for vaccine efficacy prior to testing by challenge with fully virulent Type A *F. tularensis*. Using carefully selected conditions, we then compared the relative activity of splenocytes from differentially vaccinated mice in an *in vitro* tissue culture system that measures reduction of intramacrophage bacterial numbers by immune T cells, and found that the relative strength of *in vivo* protection was clearly reflected by the relative activity of immune splenocytes *in vitro* (Figure 3). The results therefore support the utility of the co-culture assay as both a relevant functional assay in its own right.

Further, despite multiple attempts to identify strong T cell antigens involved in murine responses to *Francisella* and develop associated reagents [62]–[64], tools such as tetramers remain lacking. Studies in mice and humans suggest that host responses do not involve a classical “immunodominant” protein but are directed to a large collection of protein antigens [65]–[67]; thus although tetramer analyses may no doubt eventually prove helpful, such approaches may detect only a small fraction of the total anti-*Francisella* T cell response. Because only LVS-immune are active in specifically controlling intramacrophage growth of the homologous bacteria [25]–[26], the results presented here validate that the *in vitro* co-culture assay is a new, and currently the only available, approach to establish the relative frequency of *Francisella*-specific memory T cells in a mixed population (Figure 3, Figure S4).

The *in vitro* co-culture assay was previously developed using both *Francisella* LVS and *M. tuberculosis* as a research model to explore mechanisms of interactions between infected host macrophages and immune T lymphocytes. In many respects, this system faithfully reflects known *in vivo* T cell effector mechanisms, including both IFN-γ-dependent and non-IFN-dependent mechanisms [25]–[27], [29]–[31]. However, it should be noted that studies demonstrated that T cells from LVS-vaccinated IL-12 knockout mice are quite active in co-cultures, despite the fact that IL-12 knockout mice do not clear a vaccinating LVS infection [31]. Elsewhere, this co-culture approach was recently applied by our colleagues to murine studies of a panel of vaccine candidates for *M. tuberculosis* [68]–[69]. In those studies, the *in vitro* assay successfully discriminated between vaccines with high or moderate activity, as defined by *in vivo* protection.

Although we find the *in vitro* co-culture approach promising, as well as potentially feasible in the near term, cell-based assays are difficult to implement for human clinical trials. We therefore pursued an additional strategy, by searching for genes whose differential expression was related to the hierarchy of vaccine-induced *in vivo* protection and *in vitro* cellular activity. We focused on screening immunologically-related genes. This approach is obviously biased toward analyzing known entities instead of discovering new ones, but it offered the potential advantage of identifying relevant mediators. Remarkably, cells recovered from *in vitro* culture differentially expressed mediators at the mRNA level (Figure 4; Figure 5; Table S2) and a number of candidates emerged (Figure 4; Table 1). Of note, among these mediators, were IFN-γ and TNF-α, as might be expected, and thus validating the overall approach.

It should be noted that we observed considerable variability in mRNA levels between experiments. For example, expression of IFN-γ was always highly up-regulated in cells obtained from mice vaccinated with LVS and LVS-G, but the fold change compared to naive cells varied between about 13 and 360 (Table 1; Table S2). We suspect that biological, in addition to technical, reasons contribute to the observed variability. To increase confidence in predictors, quantifying a panel of genes is therefore likely to be preferable over assessing a single gene mediator, even an important one such as IFN-γ. This point may be especially germane to clinical settings that lack the advantages offered by using genetically identical inbred mice.

Despite the quantitative variability, from a group of about 84 genes, 16 proved to be robust enough to yield significant correlations between the magnitude of mRNA expression and survival, as well as exhibit relatively large differences in the degree of expression (Figure 5; Table 1; Figure S3; Tables S3 – S6). These likely include those whose gene products contribute directly to mechanisms, and those that are co-regulated and only epiphenomena. We are most interested in those that are mechanistically relevant, and thus likely to serve as definitive predictors across variables such as time after vaccination, route, dose, tissues sampled, and especially different animal species. Notably, the most useful of the expression differences involved up-regulated genes, which is appealing in potentially reflecting a requirement for production of a mediator to provide a particular function during challenge. It is striking that several of the leading candidates are plausibly related to Th1 cell biology, including T-bet and IFN-γ. Although TNF-α also exhibited significant differential regulation and is clearly relevant, production of TNF-α is tightly regulated to avoid toxicity, and thus *ex vivo* measurements may not be among the most useful. IL-12rβ2 is only found as part of the complete receptor for IL-12 p70, which is not expressed on resting T cells but induced by T cell activation and contributes directly to Th1 lineage commitment [70]. For example, in naive transgenic CD4^+^ T cells, IFN-γ stimulation up-regulates expression of T-bet in a STAT-1 dependent manner and promotes IL-12Rβ2 chain expression [71]. Notably, STAT-1 was also among our candidate genes with significant associations, albeit one that exhibited considerable variability and thus was not included in our two highest priority groups (Table 1).

In contrast to IFN-γ, T-bet, and IL-12rβ2, other high priority candidates such as IL-6, SOCS-1, and IL-18bp were more surprising. IL-6 has a wide variety of sources and functions, but in adaptive immunity is most commonly associated with promoting B cell activation and IgA production and infrequently with resistance to intracellular pathogens [72]. In the *Francisella* infection model, our preliminary results indicate that IL-6 knockout mice are severely impaired in their ability to survive primary LVS vaccination (Kurtz and Elkins, manuscript in preparation), as are T-bet knockout mice and IL-12rβ2 knockout mice (Melillo and Elkins, manuscript in preparation). The specific contribution of SOCS-1, an important member of a large family of “suppressor of cytokine signaling” mediators that regulate T cell differentiation as well as T cell effector functions, awaits further study. Perhaps the most unexpected candidate is IL-18 binding protein (IL-18bp); although induced by IFN-γ, its production is usually associated with cells other than leukocytes [73], and to our knowledge has no reported direct link to Th1 T cell effector functions.

Taken together, the results presented here are in important step toward the identification of T cell functions and products required for survival of lethal exposure of intracellular bacteria. We propose that the candidates described as Group 1 and Group 2 (Table 1) receive high priority for detailed direct exploration, initially in animal studies, of biological relevance and mechanistic contribution. Knowledge obtained by *in vitro* and pre clinical studies will be the key to facilitating design of assays with formats amenable to clinical studies, such as *ex vivo* re-stimulation of human peripheral blood leukocytes. It is likely that defining groups of mediators will be preferable in order to overcome issues related to variability in measurements, and ensure predictive confidence for human clinical trials. The larger goal will therefore be to establish protective levels of each individual mediator, and thus select combinations that reliably predict successful vaccination against intracellular pathogens.

## Supporting Information

Figure S1 **LVS-immune splenocytes control of intramacrophage LVS-growth within two days of co-culture.** BMMØs from C57BL/6J mice were infected with LVS at an MOI of 1:20 (bacterium-to-macrophage ratio), and co-cultured with splenocytes obtained from either naive C57BL/6J mice (“Naive,” black bars) or C57BL/6J mice infected intradermally with LVS 6 weeks previously (LVS-immune mice, “LVS,” gray bars). On the indicated days after infection, BMMØ were washed, lysed, and plated to determine the recovery of intracellular bacteria. Values shown are the mean numbers of CFU/ml ± SD of viable bacteria for triplicate samples; * indicates values significantly different between naive and LVS-immune cells (p = 0.0074 for Day 2; p = 0.001 for Day 3). Results shown are from one representative experiment of three independent experiments of similar design with similar outcome.
(TIF)

Figure S2 **Relative gene expression in LVS-immune splenocytes or LVS-infected macrophages on day two of co-culture.** BMMØs from C57BL/6J mice were either uninfected (“Mac”) or infected with LVS at an MOI of 1∶20 (bacterium-to-macrophage ratio; “Mac/LVS”), and co-cultured with splenocytes obtained from either naive C57BL/6J mice (“+Naive spleen”) or C57BL/6J mice infected intradermally with LVS 6 weeks previously (“+Primed spleen”). After two days of co-culture, non-adherent splenocytes and adherent infected macrophages were recovered from triplicate wells, pooled, mRNA prepared, and relative expression of IL-6, IFN-γ, TNF-α, and IL-12 p40 (as labeled) and quantitated using specific primer/probe sets by qRT-PCR. Values shown indicate the relative quantification of each gene of interest, as indicated by the panel label. Results shown are from one representative experiment of four independent experiments of similar design with similar outcome.
(TIF)

Figure S3 **Cytokine secretion during co-culture of LVS-infected macrophages with LVS-immune splenocytes.** BMMØs from C57BL/6J mice were either uninfected (“Mac”) or infected with LVS at an MOI of 1∶20 (bacterium-to-macrophage ratio; “Mac/LVS”) and co-cultured with splenocytes obtained from either naive C57BL/6J mice (“+Naive Splenocytes”) or C57BL/6J mice infected intradermally with LVS 6 weeks previously (“+Primed Splenocytes”). On the indicated days, supernatants from triplicate samples (obtained from LVS co-cultures immediately prior to macrophage lysis) from the indicated cultures were assessed by ELISA for IL-6 protein, TNF-α, IFN-γ, or IL-12 p40. Values shown are the mean amounts of protein ng/ml or pg/ml, as indicated, ± SD for triplicate samples. Results shown are from one representative experiment of four independent experiments of similar design with similar outcome.
(TIF)

Figure S4 **Relationship between vaccine-immune cell concentration and control of intramacrophage bacterial growth.** Data from the experiment depicted in Figure 3 were analyzed by logistic regression as described, in the text. Results shown depict log_10_ recovered bacterial CFU as a function of the concentration of cells from each vaccinated group added to co-cultures, where 1 on the X axis corresponds to 5 10^6^ splenocytes per well (see Figure 3A). ----○----, cells from LVS-vaccinated mice; - - - Δ - - - cells from LVS-G vaccinated mice; and ----+---, cells from LVS-R-vaccinated mice. Of note, separate initial analyses established that the slopes of all interpolated lines were not significantly different.
(TIF)

Table S1 **Changes in proportions of cell subpopulations over time in culture.** BMMØs from wild type C57BL/6J mice were infected with LVS at an MOI of 1∶20 (bacterium-to-macrophage ratio), and co-cultured with splenocytes obtained from either naive C57BL/6J mice or C57BL/6J mice infected intradermally with LVS 6 weeks previously (LVS-immune mice). On the indicated days after infection, non-adherent cells were recovered and pooled from triplicate co-cultures, counted under trypan blue, stained with a panel of fluorescent antibodies to cell surface markers as well as with a fluorescent viability dye, and analyzed by multi-parameter flow cytometry. ^*^ The total numbers of viable cells per well, as assessed by exclusion of trypan blue, are shown. ^†^ The proportions of gated cells, as a percent of the total viable recovered cells, are shown. Results shown are from one representative experiment of three independent experiments of similar design with similar outcome.
(DOC)

Table S2 **Relative gene expression in splenocytes derived from differentially vaccinated mice.** * In each experiment, 5 mice per vaccine group as well as 5 naive mice were challenged with 5×10^5^ - 10^6^ CFU LVS IP (determined by retrospective plate count), and survival monitored for a month; all naive mice died within 6 days of challenge, and the numbers of surviving mice within each vaccine group are shown. ‡ Gene expression was evaluated by RT PCR using TH1-TH2-TH3 panel (“Profiler”); selected genes were further evaluated using specific primer/probe sets (“qRT-PCR”). ∼All values are calculated fold differences of gene expression between vaccinated and non-vaccinated (naive) splenocytes. % In experiment 2, the same mRNA preparation was subjected to two independent PCR reactions (2a and 2b). # Mice vaccinated in experiments 2, 3, 4 and 6 were challenged, and spleens obtained for overlay and mRNA analyses, at ∼6 weeks after vaccination, and mice vaccinated in experiments 1, 5 and 7 were analyzed and challenged ∼12 weeks after vaccination. Exps 4 and 5 were vaccinated at the same time, and Exps 6 and 7 vaccinated at the same time.
(XLS)

Table S3 **Summary of all univariate logistic models using Profiler array data.** The table shows the estimated coefficient (Coef), standard error (SE), P values (P), and Akaike information criterion (AIC) for the univariate logistic regression for each gene. Here, AIC is defined as 2*k-2*Lik, where k is the number of parameters in a logistic regression model and Lik is log likelihood. In the case of univariate logistic regression, k = 2 (intercept and slope). The p value is a test as to whether relative expression of the selected gene has any effect on survival (e.g., as illustrated graphically in Figure 5). Please note that the numbers of data points available for these gene expression data are different for different genes (see Table S1, “Profiler array” data). Thus, the AIC and Lik values of different genes cannot be compared directly. For example, the univariate model with IL23a as the covariate yields the smallest AIC and largest log likelihood. The fitted model, however, is based on only 4 data points in one experiment (Table S1, Experiment 6).
(DOC)

Table S4 **Summary of all univariate logistic models using qRT-PCR data.** Similar to Table S2, this table shows the estimated coefficient (Coef), standard error (SE), P values (P), and Akaike information criterion (AIC) for the univariate logistic regression for each gene. Here, AIC is defined as 2*k-2*Lik, where k is the number of parameters in a logistic regression model and Lik is log likelihood. In the case of univariate logistic regression, k = 2 (intercept and slope). The p value is a test as to whether relative expression of the selected gene has any effect on survival (e.g., as illustrated graphically in Figure 5). Here, the numbers of data points available for these gene expression data are the same for different genes (see Table S1, “qRT-PCR” data), and thus, the AIC and Lik values of different genes may be compared directly.
(DOC)

Table S5 **Pairwise correlation coefficient for all cytokines analyzed by qRT-PCR.** Using qRT-PCR data only (Table S1, “qRT-PCR”), this table presents Pearson's correlation coefficients of standardized scores of expression level for all possible pairs of genes. Correlation coefficients greater than 0.8, suggesting a significant relationship between the relative degrees of expression of two genes in question, are **boldfaced.**
(DOC)

Table S6 **Logistic regressions for all possible pairs of genes.** Using qRT-PCR data only (Table S1, “qRT-PCR;” univariate results shown in Table S3), this table shows the estimated coefficient (Coef), standard error (SE), P values (P), and Akaike information criterion (AIC) for all possible pairs of predictors as calculated by logistic regression. The p value is a test as to whether relative expression levels of both selected genes have any effect on survival.
(DOC)

Text S1 **Optimization of conditions for analyses.**
(DOC)
