# Supplementary figures and images for: Development of Functional and Molecular Correlates of Vaccine-Induced Protection for a Model Intracellular Pathogen, F. tularensis LVS
Source: PLoS Pathog. 2012 Jan 19;8(1):e1002494. doi: 10.1371/journal.ppat.1002494 (PMC3262015; doi:10.1371/journal.ppat.1002494)

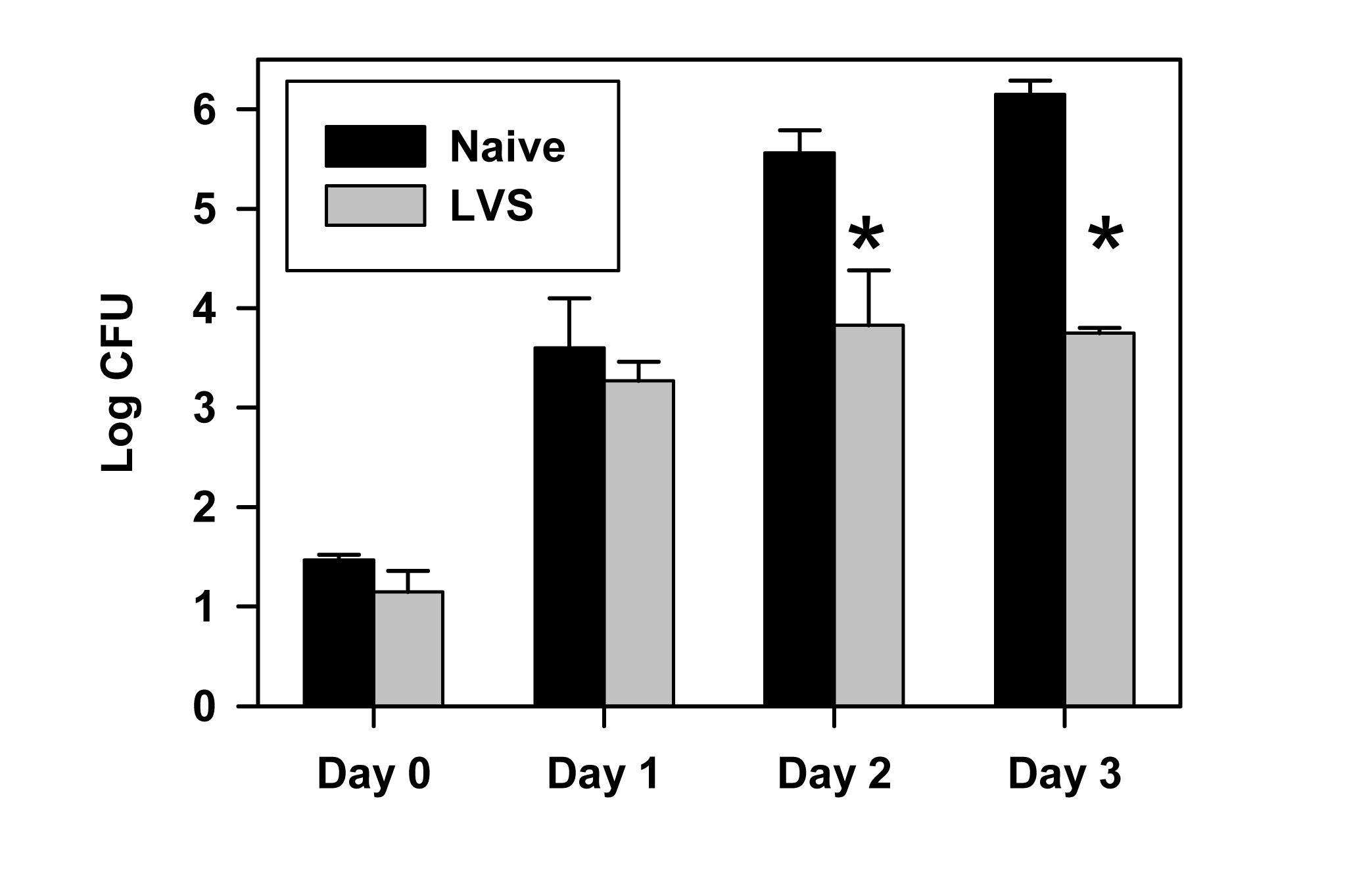

Supplement: Figure S1 — LVS-immune splenocytes control of intramacrophage LVS-growth within two days of co-culture. BMMØs from C57BL/6J mice were infected with LVS at an MOI of 1:20 (bacterium-to-macrophage ratio), and co-cultured with splenocytes obtained from either naive C57BL/6J mice (“Naive,” black bars) or C57BL/6J mice infected intradermally with LVS 6 weeks previously (LVS-immune mice, “LVS,” gray bars). On the indicated days after infection, BMMØ were washed, lysed, and plated to determine the recovery of intracellular bacteria. Values shown are the mean numbers of CFU/ml ± SD of viable bacteria for triplicate samples; * indicates values significantly different between naive and LVS-immune cells (p = 0.0074 for Day 2; p = 0.001 for Day 3). Results shown are from one representative experiment of three independent experiments of similar design with similar outcome. (TIF) [file ppat.1002494.s001.tif]

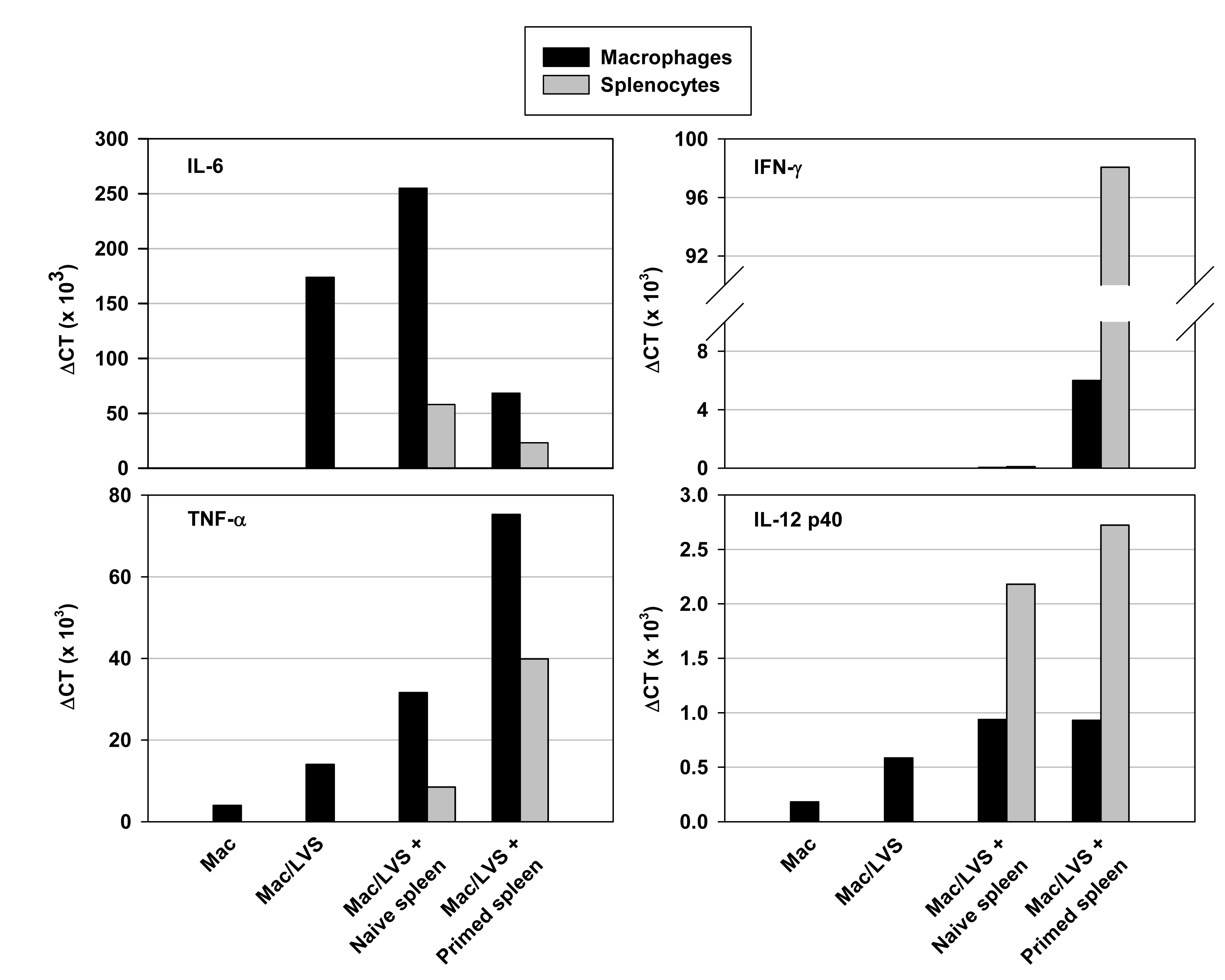

Supplement: Figure S2 — Relative gene expression in LVS-immune splenocytes or LVS-infected macrophages on day two of co-culture. BMMØs from C57BL/6J mice were either uninfected (“Mac”) or infected with LVS at an MOI of 1∶20 (bacterium-to-macrophage ratio; “Mac/LVS”), and co-cultured with splenocytes obtained from either naive C57BL/6J mice (“+Naive spleen”) or C57BL/6J mice infected intradermally with LVS 6 weeks previously (“+Primed spleen”). After two days of co-culture, non-adherent splenocytes and adherent infected macrophages were recovered from triplicate wells, pooled, mRNA prepared, and relative expression of IL-6, IFN-γ, TNF-α, and IL-12 p40 (as labeled) and quantitated using specific primer/probe sets by qRT-PCR. Values shown indicate the relative quantification of each gene of interest, as indicated by the panel label. Results shown are from one representative experiment of four independent experiments of similar design with similar outcome. (TIF) [file ppat.1002494.s002.tif]

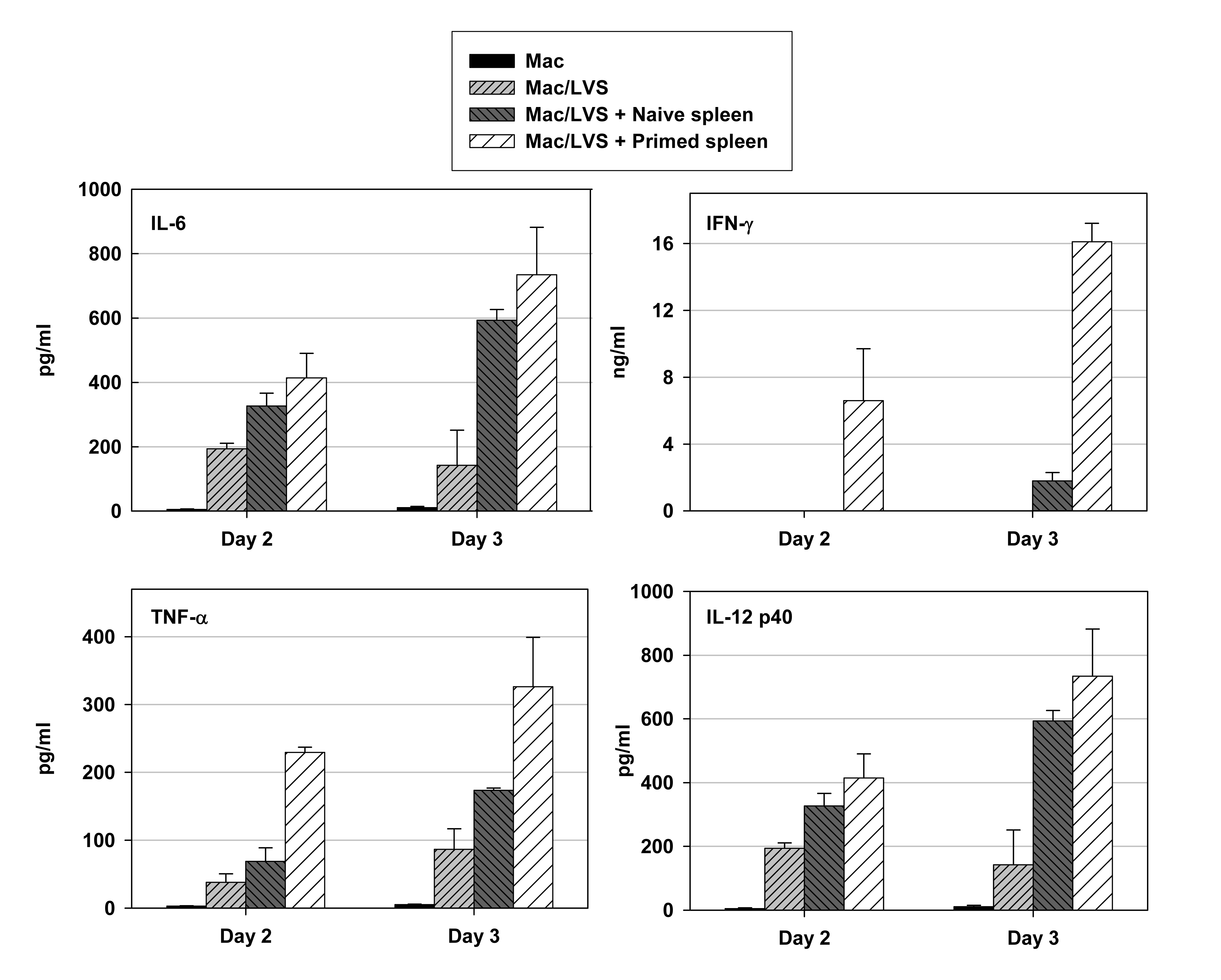

Supplement: Figure S3 — Cytokine secretion during co-culture of LVS-infected macrophages with LVS-immune splenocytes. BMMØs from C57BL/6J mice were either uninfected (“Mac”) or infected with LVS at an MOI of 1∶20 (bacterium-to-macrophage ratio; “Mac/LVS”) and co-cultured with splenocytes obtained from either naive C57BL/6J mice (“+Naive Splenocytes”) or C57BL/6J mice infected intradermally with LVS 6 weeks previously (“+Primed Splenocytes”). On the indicated days, supernatants from triplicate samples (obtained from LVS co-cultures immediately prior to macrophage lysis) from the indicated cultures were assessed by ELISA for IL-6 protein, TNF-α, IFN-γ, or IL-12 p40. Values shown are the mean amounts of protein ng/ml or pg/ml, as indicated, ± SD for triplicate samples. Results shown are from one representative experiment of four independent experiments of similar design with similar outcome. (TIF) [file ppat.1002494.s003.tif]

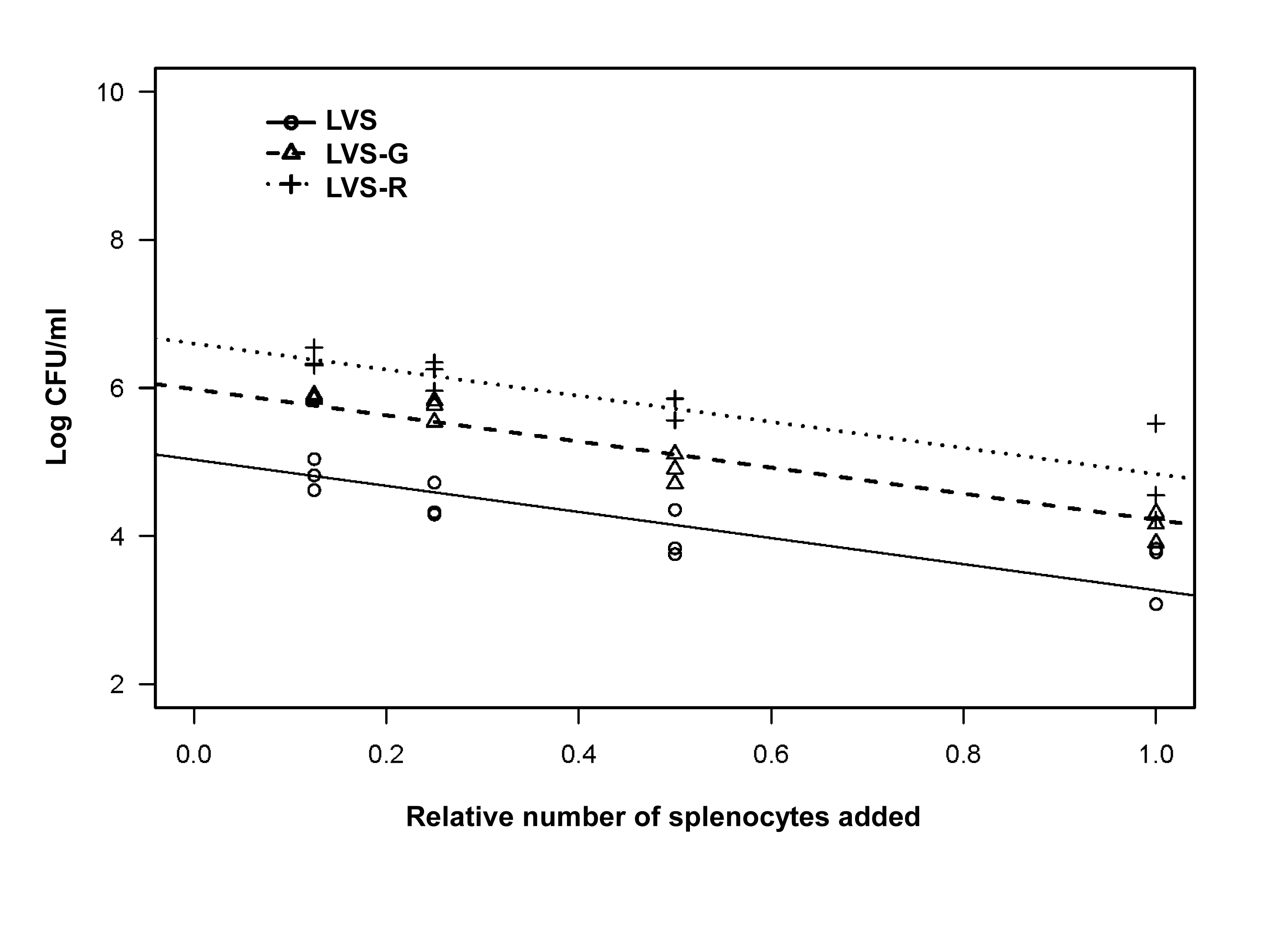

Supplement: Figure S4 — Relationship between vaccine-immune cell concentration and control of intramacrophage bacterial growth. Data from the experiment depicted in Figure 3 were analyzed by logistic regression as described, in the text. Results shown depict log10 recovered bacterial CFU as a function of the concentration of cells from each vaccinated group added to co-cultures, where 1 on the X axis corresponds to 5 106 splenocytes per well (see Figure 3A). ----○----, cells from LVS-vaccinated mice; - - - Δ - - - cells from LVS-G vaccinated mice; and ----+---, cells from LVS-R-vaccinated mice. Of note, separate initial analyses established that the slopes of all interpolated lines were not significantly different. (TIF) [file ppat.1002494.s004.tif]
