# Supplementary material for: Development of Functional and Molecular Correlates of Vaccine-Induced Protection for a Model Intracellular Pathogen, F. tularensis LVS
Source: PLoS Pathog. 2012 Jan 19;8(1):e1002494. doi: 10.1371/journal.ppat.1002494 (PMC3262015; doi:10.1371/journal.ppat.1002494)
Supplement: Table S1 — Changes in proportions of cell subpopulations over time in culture. BMMØs from wild type C57BL/6J mice were infected with LVS at an MOI of 1∶20 (bacterium-to-macrophage ratio), and co-cultured with splenocytes obtained from either naive C57BL/6J mice or C57BL/6J mice infected intradermally with LVS 6 weeks previously (LVS-immune mice). On the indicated days after infection, non-adherent cells were recovered and pooled from triplicate co-cultures, counted under trypan blue, stained with a panel of fluorescent antibodies to cell surface markers as well as with a fluorescent viability dye, and analyzed by multi-parameter flow cytometry. * The total numbers of viable cells per well, as assessed by exclusion of trypan blue, are shown. † The proportions of gated cells, as a percent of the total viable recovered cells, are shown. Results shown are from one representative experiment of three independent experiments of similar design with similar outcome. (DOC) [file ppat.1002494.s005.doc]

**Table S1**

Changes in proportions of cell subpopulations over time in culture

| **Cell type** | | **Day 0** | | **Day 1** | | **Day 2** | | **Day 3** | |
| --- | --- | --- | --- | --- | --- | --- | --- | --- | --- |
|  | | **Naive** | **LVS** | **Naive** | **LVS** | **Naive** | **LVS** | **Naive** | **LVS** |
| Total recovered viable cells | | 5x10^6^**^*^** | 5x10^6^ | 5x10^6^ | 4x10^6^ | 4.8x10^6^ | 2.4x10^6^ | 3.6x10^6^ | 1.4x10^6^ |
| Live cells | | 79^†^ | 79 | 64 | 69 | 64 | 69 | 53 | 55 |
| B cells | CD19^+^ B220^+^ | 36 | 34 | 27 | 28 | 36 | 26 | 39 | 24 |
| T cells | Thy1.2^+^ TCR^+^ | 37 | 42 | 62 | 60 | 53 | 69 | 52 | 71 |
|  | CD4^+^ | 21 | 24 | 33 | 33 | 27 | 37 | 28 | 38 |
|  | CD8^+^ | 14 | 15 | 24 | 23 | 23 | 28 | 21 | 29 |
| NK cells | DX5^+^NK1.1^+^ | 4.4 | 2.2 | 3.2 | 1.7 | 1.2 | 0.6 | 0.6 | 0.6 |
| DCs | B220^+/-^ CD11c^+^ | 2.6 | 2.8 | 1.6 | 0.9 | 1.3 | 0.6 | 1.2 | 0.3 |
| PMNs | Gr1^+^ CD11b^+^ | 1.8 | 2.0 | 0.2 | 0.5 | 0.1 | 0.1 | 0 | 0 |
| Macs | Gr1^-^ CD11b^+^ | 5.4 | 4.2 | 0.7 | 0.4 | 0.4 | 0.1 | 0.5 | 0.1 |

BMMØs from wild type C57BL/6J mice were infected with LVS at an MOI of 1:20 (bacterium-to-macrophage ratio), and co-cultured with splenocytes obtained from either naive C57BL/6J mice or C57BL/6J mice infected intradermally with LVS 6 weeks previously (LVS-immune mice). On the indicated days after infection, non-adherent cells were recovered and pooled from triplicate co-cultures, counted under trypan blue, stained with a panel of fluorescent antibodies to cell surface markers as well as with a fluorescent viability dye, and analyzed by multi-parameter flow cytometry.

**^*^** The total numbers of viable cells per well, as assessed by exclusion of trypan blue, are shown.

^†^ The proportions of gated cells, as a percent of the total viable recovered cells, are shown. Results shown are from one representative experiment of three independent experiments of similar design with similar outcome.
