# Supplementary material for: Development of Functional and Molecular Correlates of Vaccine-Induced Protection for a Model Intracellular Pathogen, F. tularensis LVS
Source: PLoS Pathog. 2012 Jan 19;8(1):e1002494. doi: 10.1371/journal.ppat.1002494 (PMC3262015; doi:10.1371/journal.ppat.1002494)
Supplement: Table S3 — Summary of all univariate logistic models using Profiler array data. The table shows the estimated coefficient (Coef), standard error (SE), P values (P), and Akaike information criterion (AIC) for the univariate logistic regression for each gene. Here, AIC is defined as 2*k-2*Lik, where k is the number of parameters in a logistic regression model and Lik is log likelihood. In the case of univariate logistic regression, k = 2 (intercept and slope). The p value is a test as to whether relative expression of the selected gene has any effect on survival (e.g., as illustrated graphically in Figure 5). Please note that the numbers of data points available for these gene expression data are different for different genes (see Table S1, “Profiler array” data). Thus, the AIC and Lik values of different genes cannot be compared directly. For example, the univariate model with IL23a as the covariate yields the smallest AIC and largest log likelihood. The fitted model, however, is based on only 4 data points in one experiment (Table S1, Experiment 6). (DOC) [file ppat.1002494.s007.doc]

**Table S3: Summary of all univariate logistic models using Profiler array data**

| Coef SE P AIC  IL17a 1.269 0.356 0.000 49.39  Bcl6 0.022 0.292 0.939 67.14  Ccl5 1.250 0.400 0.002 55.08  Ccl7 2.242 0.536 0.000 31.37  Ccr2 -1.740 0.492 0.000 44.99  Ccr3 0.420 0.376 0.264 65.86  Ccr4 0.501 0.477 0.293 66.02  Ccr5 1.302 0.404 0.001 52.71  Cd28 -3.306 0.846 0.000 37.06  Cd4 -0.417 0.301 0.166 65.20  IL23a -1.245 0.654 0.057 16.28  Cd80 1.325 0.410 0.001 52.69  Cd86 1.779 0.451 0.000 41.49  Cebpb 1.361 0.454 0.003 54.17  Crebbp -0.449 0.323 0.164 65.00  Csf2m1 1.493 0.387 0.000 45.50  Ctla4 -0.003 0.381 0.994 67.14  Cxcr3 0.543 0.493 0.271 65.89  Gata3 -1.078 0.372 0.004 57.37  Gfi1 0.390 0.333 0.242 65.70  Ccr10 0.241 0.324 0.457 51.22  Icos 0.520 0.400 0.194 65.34  Ifng 1.929 0.442 0.000 34.65  Igsf6 -0.066 0.319 0.837 67.10  IL10 0.306 0.381 0.421 66.49  IL12b -1.058 0.406 0.009 58.37  IL12rb2 1.652 0.442 0.000 45.10  IL13 -0.110 0.327 0.736 67.03  IL13ra1 0.537 0.306 0.080 63.92  IL15 1.071 0.535 0.045 62.52  IL18 0.237 0.465 0.610 66.88  IL18bp 2.474 0.563 0.000 29.17  IL18r1 -0.283 0.255 0.268 65.89  ILr1 0.262 0.376 0.487 66.66  IL2 0.452 0.330 0.170 65.16  IL27 3.315 1.117 0.003 24.75  IL27ra -0.876 0.349 0.012 59.79  IL2ra 0.967 0.352 0.006 58.63  IL4 -0.484 0.340 0.155 46.84  IL4ra -0.523 0.309 0.090 64.15  IL5 -0.302 0.291 0.299 60.94 | Coef SE P AIC    IL6 1.876 0.423 0.000 33.69  IL7 -0.244 0.286 0.394 66.40  IL9 -0.449 0.614 0.465 20.04  Inha 0.048 0.304 0.875 59.14  Irf1 1.627 0.414 0.000 41.90  Irf4 -0.414 0.334 0.214 65.55  Jak1 -2.353 0.663 0.000 45.32  Jak2 -0.184 0.268 0.494 66.68  Jak3 0.066 0.380 0.861 67.11  Junb 0.355 0.348 0.308 66.08  Maf -1.481 0.489 0.002 53.23  Mapk8 -0.242 0.261 0.353 66.27  Mapk9 0.425 0.377 0.259 65.82  Nfatc1 -0.218 0.256 0.394 66.41  Nfatc2 -0.614 0.372 0.099 64.15  Nfatc2ip -0.390 0.263 0.139 64.87  Nfatc3 -2.042 0.617 0.001 52.91  Nfkb1 -0.446 0.327 0.173 65.24  Ptprc 0.262 0.327 0.424 66.50  Pcgf2 0.362 0.257 0.160 65.11  Socs1 1.933 0.423 0.000 35.29  Socs3 1.200 0.429 0.005 56.16  Socs5 -0.717 0.339 0.034 61.71  Spp1 -0.100 0.303 0.743 67.04  Stat1 1.505 0.406 0.000 47.94  Stat4 -1.468 0.504 0.004 32.48  Tbx21 2.192 0.523 0.000 35.44  Tcfcp2 -0.603 0.278 0.030 62.11  Tgfb3 -0.542 0.293 0.064 63.49  Tlr4 -0.605 0.315 0.055 63.29  Tlr6 0.681 0.310 0.028 61.74  Tmed1 1.886 0.577 0.001 46.66  Tnf 1.416 0.370 0.000 46.17  Tnfrsf4 1.170 0.463 0.011 59.77  CD40 1.834 0.502 0.000 47.82  CD27 -1.623 0.427 0.000 47.54  Tnfrsf8 0.379 0.262 0.147 64.97  Tnfsf4 1.367 0.512 0.008 58.82  CD40lg -0.996 0.335 0.003 56.14  Tyk2 -1.336 0.408 0.001 51.88  Yy1 -0.974 0.339 0.004 57.84 |
| --- | --- |

The table shows the estimated coefficient (Coef), standard error (SE), P values (P), and Akaike information criterion (AIC) for the univariate logistic regression for each gene. Here, AIC is defined as 2*k-2*Lik, where k is the number of parameters in a logistic regression model and Lik is log likelihood. In the case of univariate logistic regression, k=2 (intercept and slope). The p value is a test as to whether relative expression of the selected gene has any effect on survival (e.g., as illustrated graphically in Figure 5).

Please note that the numbers of data points available for these gene expression data are different for different genes (see Table S1, “Profiler array” data). Thus, the AIC and Lik values of different genes cannot be compared directly. For example, the univariate model with IL23a as the covariate yields the smallest AIC and largest log likelihood. The fitted model, however, is based on only 4 data points in one experiment (Table S1, Experiment 6).
