# Supplementary material for: Development of Functional and Molecular Correlates of Vaccine-Induced Protection for a Model Intracellular Pathogen, F. tularensis LVS
Source: PLoS Pathog. 2012 Jan 19;8(1):e1002494. doi: 10.1371/journal.ppat.1002494 (PMC3262015; doi:10.1371/journal.ppat.1002494)
Supplement: Table S4 — Summary of all univariate logistic models using qRT-PCR data. Similar to Table S2, this table shows the estimated coefficient (Coef), standard error (SE), P values (P), and Akaike information criterion (AIC) for the univariate logistic regression for each gene. Here, AIC is defined as 2*k-2*Lik, where k is the number of parameters in a logistic regression model and Lik is log likelihood. In the case of univariate logistic regression, k = 2 (intercept and slope). The p value is a test as to whether relative expression of the selected gene has any effect on survival (e.g., as illustrated graphically in Figure 5). Here, the numbers of data points available for these gene expression data are the same for different genes (see Table S1, “qRT-PCR” data), and thus, the AIC and Lik values of different genes may be compared directly. (DOC) [file ppat.1002494.s008.doc]

**Table S4: Summary of all univariate logistic models using qRT-PCR data**

| **Coef SE P AIC**  **Ifng 2.327 0.449 0.000 33.27**  **Tnf 1.797 0.485 0.000 60.16**  **IL6 2.311 0.529 0.000 42.90**  IL12b -0.445 0.252 0.078 80.97  **IL12rb2 2.545 0.519 0.000 36.94**  **IL17a 0.669 0.262 0.011 77.10**  **IL18bp 1.842 0.431 0.000 51.44**  **IL23a 0.869 0.300 0.004 74.01**  **IL27 1.559 0.355 0.000 55.98**  IL27ra 0.065 0.240 0.787 84.16  **Csf2 1.775 0.386 0.000 50.83**  **Tbx21 2.012 0.477 0.000 50.91**  IL13 0.407 0.259 0.116 81.60  **Ccl7 1.155 0.295 0.000 65.30**  Ccr2 -0.281 0.241 0.243 82.86  Ccr3 -0.189 0.238 0.428 83.61  **Ccr5 1.177 0.312 0.000 65.94**  Gata3 -0.360 0.245 0.141 82.03  **Irf1 1.600 0.356 0.000 53.54**  **Socs1 1.903 0.393 0.000 45.60**  **Stat1 0.992 0.285 0.001 69.75**  **IL22 2.083 0.457 0.000 44.20** |
| --- |

Similar to Table S2, this table shows the estimated coefficient (Coef), standard error (SE), P values (P), and Akaike information criterion (AIC) for the univariate logistic regression for each gene. Here, AIC is defined as 2*k-2*Lik, where k is the number of parameters in a logistic regression model and Lik is log likelihood. In the case of univariate logistic regression, k=2 (intercept and slope). The p value is a test as to whether relative expression of the selected gene has any effect on survival (e.g., as illustrated graphically in Figure 5).

Here, the numbers of data points available for these gene expression data are the same for different genes (see Table S1, “qRT-PCR” data), and thus, the AIC and Lik values of different genes may be compared directly.
