# Supplementary material for: Development of Functional and Molecular Correlates of Vaccine-Induced Protection for a Model Intracellular Pathogen, F. tularensis LVS
Source: PLoS Pathog. 2012 Jan 19;8(1):e1002494. doi: 10.1371/journal.ppat.1002494 (PMC3262015; doi:10.1371/journal.ppat.1002494)
Supplement: Table S5 — Pairwise correlation coefficient for all cytokines analyzed by qRT-PCR. Using qRT-PCR data only (Table S1, “qRT-PCR”), this table presents Pearson's correlation coefficients of standardized scores of expression level for all possible pairs of genes. Correlation coefficients greater than 0.8, suggesting a significant relationship between the relative degrees of expression of two genes in question, are boldfaced. (DOC) [file ppat.1002494.s009.doc]

**Table S5: Pairwise correlation coefficient for all cytokines analyzed by qRT-PCR**

| Ifng Tnf Il6 Il12b Il12rb2 Il17a Il18bp

-------------+---------------------------------------------------------------

Ifng | 1.0000

Tnf | 0.6403 1.0000

Il6 | **0.8497 0.8230**  1.0000

Il12b | -0.2126 0.3211 0.0668 1.0000

Il12rb2 |  **0.8656 0.8448** 0.7955 0.0747 1.0000

Il17a | 0.4985 0.4357 0.7233 0.0096 0.3200 1.0000

Il18bp | 0.7878 **0.8017**  **0.8889** 0.2064 **0.8041** 0.5377 1.0000

Il23a | 0.4661 0.7353 0.4259 0.3660 0.7925 0.0516 0.4500

Il27 | 0.7161 **0.8727**  **0.8463** 0.2639 **0.8859** 0.5178 **0.8502**

Il27ra | 0.1314 0.3075 -0.0601 0.3632 0.3890 -0.3479 0.2351

Csf2 | **0.8409** 0.6618 0.6153 0.0408 **0.8944** 0.2028 0.6266

Tbx21 | 0.6838 0.7192 0.6510 0.1150 **0.8473** 0.1993 0.7508

Il13 | 0.2370 0.5484 0.3226 0.4576 0.5384 0.2432 0.2826

Ccl7 | 0.5992 0.6992 0.7782 0.3054 0.6640 0.5292 **0.8564**

Ccr2 | -0.2528 0.0178 -0.3306 0.5350 0.0937 -0.6289 -0.1483

Ccr3 | -0.1719 0.2780 -0.1326 0.7177 0.2055 -0.4731 0.1337

Ccr5 | 0.5037 0.6595 0.4480 0.1732 **0.8082** 0.0228 0.4201

Gata3 | -0.1689 0.0905 -0.2700 0.5462 0.1230 -0.3604 -0.1792

Irf1 |  **0.8650** 0.5257 0.6851 -0.1776 0.7175 0.4746 0.7727

Socs1 |  **0.9522** 0.5806 0.7831 -0.1677 0.7694 0.5253 0.7693

Stat1 | 0.7308 0.4600 0.5514 0.0434 0.6252 0.4331 0.7015

Il22 | **0.9160** 0.6251 **0.8459** -0.1184 **0.8130** 0.6527 0.7756

| Il23a Il27 Il27ra Csf2 Tbx21 Il13 Ccl7

-------------+---------------------------------------------------------------

Il23a | 1.0000

Il27 | 0.7116 1.0000

Il27ra | 0.6243 0.2230 1.0000

Csf2 | 0.7951 0.6831 0.5059 1.0000

Tbx21 | 0.6920 0.7558 0.5343 0.7086 1.0000

Il13 | 0.7239 0.6173 0.1773 0.5037 0.3001 1.0000

Ccl7 | 0.3796 **0.8427** -0.0083 0.4682 0.4915 0.4957 1.0000

Ccr2 | 0.5084 0.0567 0.5845 0.1884 0.1908 0.3664 -0.1312

Ccr3 | 0.5250 0.2405 0.6850 0.2356 0.2726 0.3866 0.1714

Ccr5 | **0.8702** 0.7043 0.3100 0.7304 0.6197 0.7897 0.4727

Gata3 | 0.5383 0.0206 0.5296 0.3053 0.0651 0.5944 -0.1639

Irf1 | 0.3069 0.5855 0.2499 0.7425 0.5493 0.1974 0.6042

Socs1 | 0.3866 0.6197 0.1890 **0.8218** 0.6055 0.2041 0.5646

Stat1 | 0.3355 0.5295 0.3950 0.6936 0.4205 0.2395 0.5634

Il22 | 0.4700 0.7801 0.0518 0.7768 0.5740 0.3892 0.6731

| Ccr2 Ccr3 Ccr5 Gata3 Irf1 Socs1 Stat1

-------------+---------------------------------------------------------------

Ccr2 | 1.0000

Ccr3 | **0.8445** 1.0000

Ccr5 | 0.4002 0.3601 1.0000

Gata3 | 0.7827 0.6702 0.4355 1.0000

Irf1 | -0.3195 -0.0875 0.3081 -0.1276 1.0000

Socs1 | -0.2861 -0.1539 0.3639 -0.0959 **0.9345** 1.0000

Stat1 | -0.2053 0.0452 0.2355 0.0301 **0.9196** **0.8324** 1.0000

Il22 | -0.2624 -0.1584 0.5022 -0.1168 **0.8457** **0.8875** 0.7582

Using qRT-PCR data only (Table S1, “qRT-PCR”), this table presents Pearson’s correlation coefficients of standardized scores of expression level for all possible pairs of genes. Correlation coefficients greater than 0.8, suggesting a significant relationship between the relative degrees of expression of two genes in question, are **boldfaced.**
