# Supplementary material for: Development of Functional and Molecular Correlates of Vaccine-Induced Protection for a Model Intracellular Pathogen, F. tularensis LVS
Source: PLoS Pathog. 2012 Jan 19;8(1):e1002494. doi: 10.1371/journal.ppat.1002494 (PMC3262015; doi:10.1371/journal.ppat.1002494)
Supplement: Table S6 — Logistic regressions for all possible pairs of genes. Using qRT-PCR data only (Table S1, “qRT-PCR;” univariate results shown in Table S3), this table shows the estimated coefficient (Coef), standard error (SE), P values (P), and Akaike information criterion (AIC) for all possible pairs of predictors as calculated by logistic regression. The p value is a test as to whether relative expression levels of both selected genes have any effect on survival. (DOC) [file ppat.1002494.s010.doc]

**Table S6: Logistic regressions for all possible pairs of genes**

|  |  | **Predictor 1** | | | **Predictor 2** | | |  |
| --- | --- | --- | --- | --- | --- | --- | --- | --- |
| **Predictor 1** | **Predictor 2** | Coef | SE | P | Coef | SE | P | AIC |
| Ifng | Tnf | 2.14 | 0.493 | <0.001 | 0.529 | 0.623 | 0.396 | 34.48 |
| Ifng | IL6 | 1.756 | 0.564 | 0.002 | 1.043 | 0.706 | 0.14 | 32.98 |
| Ifng | IL12b | 2.416 | 0.512 | <0.001 | 0.154 | 0.365 | 0.673 | 35.09 |
| Ifng | IL12rb2 | 1.659 | 0.822 | 0.044 | 0.828 | 0.909 | 0.362 | 34.39 |
| Ifng | IL17a | 2.35 | 0.465 | <0.001 | -0.091 | 0.465 | 0.844 | 35.23 |
| Ifng | IL18bp | 1.962 | 0.527 | <0.001 | 0.696 | 0.641 | 0.278 | 34.11 |
| Ifng | IL23a | 2.326 | 0.483 | <0.001 | 0.002 | 0.432 | 0.997 | 35.27 |
| Ifng | IL27 | 2.083 | 0.505 | <0.001 | 0.508 | 0.504 | 0.313 | 34.23 |
| Ifng | IL27ra | 2.418 | 0.471 | <0.001 | -0.408 | 0.427 | 0.339 | 34.31 |
| Ifng | Csf2 | 2.674 | 0.758 | <0.001 | -0.402 | 0.682 | 0.556 | 34.91 |
| Ifng | Tbx21 | 1.905 | 0.513 | <0.001 | 0.81 | 0.591 | 0.17 | 33.21 |
| Ifng | IL13 | 2.326 | 0.454 | <0.001 | 0.003 | 0.319 | 0.994 | 35.27 |
| Ifng | Ccl7 | 2.194 | 0.473 | <0.001 | 0.357 | 0.394 | 0.366 | 34.46 |
| Ifng | Ccr2 | 2.376 | 0.468 | <0.001 | 0.186 | 0.37 | 0.615 | 35.02 |
| Ifng | Ccr3 | 2.46 | 0.501 | <0.001 | 0.315 | 0.368 | 0.391 | 34.53 |
| Ifng | Ccr5 | 2.16 | 0.492 | <0.001 | 0.291 | 0.393 | 0.46 | 34.72 |
| Ifng | Gata3 | 2.315 | 0.449 | <0.001 | -0.236 | 0.346 | 0.494 | 34.79 |
| Ifng | Irf1 | 2.783 | 0.703 | <0.001 | -0.516 | 0.578 | 0.372 | 34.48 |
| Ifng | Socs1 | 4.033 | 1.212 | 0.001 | -1.752 | 1.095 | 0.11 | 32.52 |
| Ifng | Stat1 | 2.978 | 0.629 | <0.001 | -0.834 | 0.481 | 0.083 | 32.01 |
| Ifng | IL22 | 2.532 | 0.874 | 0.004 | -0.239 | 0.859 | 0.781 | 35.19 |
| Tnf | IL6 | -1.715 | 0.874 | 0.05 | 4.019 | 1.154 | 0 | 41.07 |
| Tnf | IL12b | 2.389 | 0.63 | <0.001 | -1.108 | 0.357 | 0.002 | 49.71 |
| Tnf | IL12rb2 | -0.229 | 0.797 | 0.774 | 2.679 | 0.708 | 0 | 38.86 |
| Tnf | IL17a | 2.036 | 0.714 | 0.004 | -0.193 | 0.394 | 0.624 | 61.92 |
| Tnf | IL18bp | 0.512 | 0.63 | 0.417 | 1.553 | 0.552 | 0.005 | 52.75 |
| Tnf | IL23a | 1.704 | 0.513 | 0.001 | 0.257 | 0.412 | 0.533 | 61.77 |
| Tnf | IL27 | 0.458 | 0.785 | 0.559 | 1.231 | 0.639 | 0.054 | 57.64 |
| Tnf | IL27ra | 1.852 | 0.555 | 0.001 | 0.084 | 0.364 | 0.817 | 62.11 |
| Tnf | Csf2 | 1.363 | 0.593 | 0.022 | 1.399 | 0.396 | 0 | 46.88 |
| Tnf | Tbx21 | 1.492 | 0.603 | 0.013 | 1.759 | 0.509 | 0.001 | 45.75 |
| Tnf | IL13 | 2.061 | 0.572 | <0.001 | -0.331 | 0.302 | 0.273 | 60.97 |
| Tnf | Ccl7 | 1.344 | 0.664 | 0.043 | 0.38 | 0.434 | 0.382 | 61.4 |
| Tnf | Ccr2 | 1.733 | 0.505 | 0.001 | -0.114 | 0.303 | 0.708 | 62.02 |
| Tnf | Ccr3 | 1.708 | 0.46 | <0.001 | -0.431 | 0.323 | 0.183 | 60.31 |
| Tnf | Ccr5 | 1.441 | 0.516 | 0.005 | 0.718 | 0.365 | 0.049 | 58.04 |
| Tnf | Gata3 | 1.664 | 0.473 | <0.001 | -0.378 | 0.3 | 0.207 | 60.57 |
| Tnf | Irf1 | 1.403 | 0.579 | 0.015 | 1.259 | 0.374 | 0.001 | 47.82 |
| Tnf | Socs1 | 1.193 | 0.605 | 0.048 | 1.656 | 0.436 | 0 | 42.72 |
| Tnf | Stat1 | 1.579 | 0.518 | 0.002 | 0.62 | 0.312 | 0.047 | 58.04 |
| Tnf | IL22 | 0.598 | 0.544 | 0.272 | 1.779 | 0.512 | 0.001 | 44.81 |
| IL6 | IL12b | 2.319 | 0.518 | <0.001 | -0.7 | 0.344 | 0.042 | 40.2 |
| IL6 | IL12rb2 | 1.042 | 0.647 | 0.107 | 1.799 | 0.652 | 0.006 | 35.94 |
| IL6 | IL17a | 3.901 | 0.892 | <0.001 | -1.638 | 0.604 | 0.007 | 35.36 |
| IL6 | IL18bp | 1.923 | 0.678 | 0.005 | 0.567 | 0.623 | 0.363 | 44.05 |
| IL6 | IL23a | 2.171 | 0.552 | <0.001 | 0.32 | 0.463 | 0.49 | 44.41 |
| IL6 | IL27 | 2.543 | 0.825 | 0.002 | -0.226 | 0.6 | 0.707 | 44.75 |
| IL6 | IL27ra | 3.003 | 0.812 | <0.001 | 0.933 | 0.555 | 0.093 | 41.35 |
| IL6 | Csf2 | 1.982 | 0.606 | 0.001 | 1.132 | 0.407 | 0.005 | 36.07 |
| IL6 | Tbx21 | 1.689 | 0.56 | 0.003 | 1.196 | 0.538 | 0.026 | 39.21 |
| IL6 | IL13 | 2.483 | 0.584 | <0.001 | -0.29 | 0.348 | 0.404 | 44.16 |
| IL6 | Ccl7 | 2.5 | 0.647 | <0.001 | -0.228 | 0.434 | 0.599 | 44.61 |
| IL6 | Ccr2 | 2.499 | 0.587 | <0.001 | 0.378 | 0.37 | 0.306 | 43.81 |
| IL6 | Ccr3 | 2.395 | 0.577 | <0.001 | 0.168 | 0.354 | 0.635 | 44.67 |
| IL6 | Ccr5 | 1.935 | 0.535 | <0.001 | 0.628 | 0.412 | 0.127 | 42.47 |
| IL6 | Gata3 | 2.378 | 0.575 | <0.001 | 0.128 | 0.36 | 0.722 | 44.77 |
| IL6 | Irf1 | 2.184 | 0.656 | 0.001 | 0.989 | 0.393 | 0.012 | 37.78 |
| IL6 | Socs1 | 1.874 | 0.653 | 0.004 | 1.269 | 0.466 | 0.007 | 36.61 |
| IL6 | Stat1 | 2.32 | 0.59 | <0.001 | 0.515 | 0.337 | 0.127 | 42.52 |
| IL6 | IL22 | 1.532 | 0.649 | 0.018 | 1.231 | 0.577 | 0.033 | 39.73 |
| IL12b | IL12rb2 | -0.525 | 0.333 | 0.115 | 2.471 | 0.519 | 0 | 36.36 |
| IL12b | IL17a | -0.476 | 0.262 | 0.069 | 0.701 | 0.271 | 0.01 | 75.59 |
| IL12b | IL18bp | -0.908 | 0.309 | 0.003 | 2.141 | 0.477 | 0 | 43.47 |
| IL12b | IL23a | -0.948 | 0.319 | 0.003 | 1.256 | 0.345 | 0 | 65.44 |
| IL12b | IL27 | -1.153 | 0.377 | 0.002 | 2.123 | 0.499 | 0 | 45.46 |
| IL12b | IL27ra | -0.559 | 0.281 | 0.047 | 0.276 | 0.267 | 0.302 | 81.89 |
| IL12b | Csf2 | -0.639 | 0.32 | 0.046 | 1.815 | 0.394 | 0 | 48.47 |
| IL12b | Tbx21 | -0.558 | 0.293 | 0.057 | 1.991 | 0.481 | 0 | 49 |
| IL12b | IL13 | -0.841 | 0.323 | 0.009 | 0.815 | 0.323 | 0.012 | 75.38 |
| IL12b | Ccl7 | -1.32 | 0.41 | 0.001 | 1.891 | 0.454 | 0 | 52.33 |
| IL12b | Ccr2 | -0.409 | 0.3 | 0.172 | -0.063 | 0.288 | 0.826 | 82.93 |
| IL12b | Ccr3 | -0.645 | 0.371 | 0.082 | 0.262 | 0.347 | 0.451 | 82.4 |
| IL12b | Ccr5 | -0.801 | 0.323 | 0.013 | 1.357 | 0.337 | 0 | 60.78 |
| IL12b | Gata3 | -0.349 | 0.299 | 0.242 | -0.171 | 0.29 | 0.555 | 82.62 |
| IL12b | Irf1 | -0.236 | 0.297 | 0.427 | 1.558 | 0.36 | 0 | 54.9 |
| IL12b | Socs1 | -0.154 | 0.316 | 0.627 | 1.847 | 0.399 | 0 | 47.36 |
| IL12b | Stat1 | -0.528 | 0.278 | 0.058 | 1.013 | 0.287 | 0 | 67.89 |
| IL12b | IL22 | -0.368 | 0.312 | 0.238 | 2.076 | 0.465 | 0 | 44.77 |
| IL12rb2 | IL17a | 2.498 | 0.538 | <0.001 | 0.118 | 0.379 | 0.756 | 38.85 |
| IL12rb2 | IL18bp | 2.144 | 0.618 | 0.001 | 0.602 | 0.575 | 0.295 | 37.83 |
| IL12rb2 | IL23a | 3.679 | 0.774 | <0.001 | -1.536 | 0.643 | 0.017 | 32.24 |
| IL12rb2 | IL27 | 2.862 | 0.785 | <0.001 | -0.388 | 0.697 | 0.578 | 38.62 |
| IL12rb2 | IL27ra | 2.585 | 0.516 | <0.001 | -0.592 | 0.415 | 0.154 | 36.79 |
| IL12rb2 | Csf2 | 2.928 | 0.915 | 0.001 | -0.372 | 0.705 | 0.597 | 38.66 |
| IL12rb2 | Tbx21 | 2.166 | 0.597 | <0.001 | 0.808 | 0.59 | 0.171 | 36.99 |
| IL12rb2 | IL13 | 3.087 | 0.638 | <0.001 | -0.747 | 0.365 | 0.041 | 34.43 |
| IL12rb2 | Ccl7 | 2.645 | 0.643 | <0.001 | -0.121 | 0.443 | 0.785 | 38.87 |
| IL12rb2 | Ccr2 | 2.56 | 0.536 | <0.001 | -0.53 | 0.403 | 0.189 | 37.06 |
| IL12rb2 | Ccr3 | 2.472 | 0.514 | <0.001 | -0.414 | 0.384 | 0.281 | 37.72 |
| IL12rb2 | Ccr5 | 3.715 | 0.936 | <0.001 | -1.098 | 0.62 | 0.076 | 35.44 |
| IL12rb2 | Gata3 | 2.691 | 0.582 | <0.001 | -0.715 | 0.371 | 0.054 | 34.82 |
| IL12rb2 | Irf1 | 2.221 | 0.611 | <0.001 | 0.397 | 0.433 | 0.359 | 38.08 |
| IL12rb2 | Socs1 | 1.923 | 0.672 | 0.004 | 0.674 | 0.528 | 0.202 | 37.23 |
| IL12rb2 | Stat1 | 2.796 | 0.638 | <0.001 | -0.3 | 0.406 | 0.459 | 38.38 |
| IL12rb2 | IL22 | 2.092 | 0.829 | 0.012 | 0.453 | 0.68 | 0.506 | 38.48 |
| IL17a | IL18bp | -0.088 | 0.353 | 0.802 | 1.888 | 0.47 | 0 | 53.38 |
| IL17a | IL23a | 0.666 | 0.267 | 0.013 | 0.889 | 0.319 | 0.005 | 69.29 |
| IL17a | IL27 | -0.239 | 0.379 | 0.528 | 1.761 | 0.499 | 0 | 57.58 |
| IL17a | IL27ra | 0.807 | 0.287 | 0.005 | 0.366 | 0.282 | 0.195 | 77.34 |
| IL17a | Csf2 | 0.572 | 0.345 | 0.097 | 1.652 | 0.371 | 0 | 49.89 |
| IL17a | Tbx21 | 0.481 | 0.299 | 0.108 | 1.945 | 0.49 | 0 | 50.19 |
| IL17a | IL13 | 0.604 | 0.269 | 0.025 | 0.246 | 0.262 | 0.347 | 78.19 |
| IL17a | Ccl7 | 0.147 | 0.326 | 0.652 | 1.075 | 0.34 | 0.002 | 67.1 |
| IL17a | Ccr2 | 0.797 | 0.332 | 0.016 | 0.199 | 0.314 | 0.525 | 78.69 |
| IL17a | Ccr3 | 0.739 | 0.296 | 0.013 | 0.145 | 0.279 | 0.603 | 78.83 |
| IL17a | Ccr5 | 0.744 | 0.291 | 0.011 | 1.25 | 0.345 | 0 | 60.67 |
| IL17a | Gata3 | 0.615 | 0.276 | 0.026 | -0.156 | 0.263 | 0.553 | 78.75 |
| IL17a | Irf1 | 0.15 | 0.335 | 0.654 | 1.546 | 0.375 | 0 | 55.34 |
| IL17a | Socs1 | 0.048 | 0.382 | 0.9 | 1.89 | 0.406 | 0 | 47.59 |
| IL17a | Stat1 | 0.385 | 0.296 | 0.194 | 0.854 | 0.3 | 0.004 | 70.02 |
| IL17a | IL22 | -0.626 | 0.466 | 0.179 | 2.45 | 0.549 | 0 | 44.23 |
| IL18bp | IL23a | 1.701 | 0.434 | <0.001 | 0.565 | 0.415 | 0.174 | 51.49 |
| IL18bp | IL27 | 1.365 | 0.603 | 0.023 | 0.575 | 0.53 | 0.278 | 52.23 |
| IL18bp | IL27ra | 1.981 | 0.463 | <0.001 | -0.478 | 0.392 | 0.223 | 51.85 |
| IL18bp | Csf2 | 1.443 | 0.497 | 0.004 | 1.249 | 0.397 | 0.002 | 42.13 |
| IL18bp | Tbx21 | 1.117 | 0.497 | 0.025 | 1.322 | 0.556 | 0.017 | 46.93 |
| IL18bp | IL13 | 1.837 | 0.445 | <0.001 | 0.013 | 0.285 | 0.965 | 53.44 |
| IL18bp | Ccl7 | 2.324 | 0.685 | 0.001 | -0.489 | 0.517 | 0.344 | 52.53 |
| IL18bp | Ccr2 | 1.843 | 0.439 | <0.001 | -0.179 | 0.296 | 0.547 | 53.08 |
| IL18bp | Ccr3 | 1.958 | 0.448 | <0.001 | -0.511 | 0.302 | 0.091 | 50.4 |
| IL18bp | Ccr5 | 1.559 | 0.42 | <0.001 | 0.897 | 0.369 | 0.015 | 46.82 |
| IL18bp | Gata3 | 1.833 | 0.441 | <0.001 | -0.22 | 0.321 | 0.492 | 52.96 |
| IL18bp | Irf1 | 1.245 | 0.525 | 0.018 | 0.847 | 0.435 | 0.051 | 49.24 |
| IL18bp | Socs1 | 1.093 | 0.525 | 0.037 | 1.422 | 0.464 | 0.002 | 42.74 |
| IL18bp | Stat1 | 1.838 | 0.503 | <0.001 | 0.007 | 0.373 | 0.986 | 53.44 |
| IL18bp | IL22 | 0.866 | 0.519 | 0.095 | 1.536 | 0.536 | 0.004 | 43.13 |
| IL23a | IL27 | -0.081 | 0.438 | 0.853 | 1.601 | 0.422 | 0 | 57.95 |
| IL23a | IL27ra | 1.27 | 0.376 | 0.001 | -0.657 | 0.334 | 0.049 | 71.89 |
| IL23a | Csf2 | -0.921 | 0.539 | 0.088 | 2.433 | 0.573 | 0 | 49.7 |
| IL23a | Tbx21 | 0.13 | 0.431 | 0.762 | 1.968 | 0.498 | 0 | 52.81 |
| IL23a | IL13 | 1.168 | 0.433 | 0.007 | -0.382 | 0.375 | 0.309 | 74.98 |
| IL23a | Ccl7 | 0.625 | 0.353 | 0.077 | 0.966 | 0.299 | 0.001 | 63.88 |
| IL23a | Ccr2 | 1.789 | 0.517 | 0.001 | -1.338 | 0.477 | 0.005 | 63.15 |
| IL23a | Ccr3 | 1.406 | 0.384 | <0.001 | -1.002 | 0.39 | 0.01 | 67.34 |
| IL23a | Ccr5 | -0.597 | 0.618 | 0.335 | 1.686 | 0.631 | 0.008 | 66.96 |
| IL23a | Gata3 | 1.845 | 0.459 | <0.001 | -1.428 | 0.392 | 0 | 59.02 |
| IL23a | Irf1 | 0.709 | 0.383 | 0.064 | 1.448 | 0.351 | 0 | 51.82 |
| IL23a | Socs1 | 0.419 | 0.391 | 0.284 | 1.756 | 0.404 | 0 | 46.43 |
| IL23a | Stat1 | 0.695 | 0.331 | 0.036 | 0.827 | 0.289 | 0.004 | 66.81 |
| IL23a | IL22 | -0.115 | 0.432 | 0.79 | 2.172 | 0.578 | 0 | 46.13 |
| IL27 | IL27ra | 1.549 | 0.352 | <0.001 | -0.075 | 0.318 | 0.814 | 57.93 |
| IL27 | Csf2 | 1.075 | 0.445 | 0.016 | 1.252 | 0.404 | 0.002 | 46.64 |
| IL27 | Tbx21 | 1.022 | 0.45 | 0.023 | 1.521 | 0.516 | 0.003 | 47.44 |
| IL27 | IL13 | 2.191 | 0.527 | <0.001 | -0.8 | 0.38 | 0.035 | 53.31 |
| IL27 | Ccl7 | 1.884 | 0.676 | 0.005 | -0.319 | 0.548 | 0.56 | 57.64 |
| IL27 | Ccr2 | 1.517 | 0.343 | <0.001 | -0.361 | 0.307 | 0.239 | 56.55 |
| IL27 | Ccr3 | 1.622 | 0.35 | <0.001 | -0.596 | 0.336 | 0.076 | 54.51 |
| IL27 | Ccr5 | 1.292 | 0.408 | 0.002 | 0.503 | 0.397 | 0.205 | 56.35 |
| IL27 | Gata3 | 1.514 | 0.348 | <0.001 | -0.411 | 0.306 | 0.179 | 56.16 |
| IL27 | Irf1 | 1.155 | 0.421 | 0.006 | 1.105 | 0.362 | 0.002 | 47.08 |
| IL27 | Socs1 | 1.041 | 0.439 | 0.018 | 1.563 | 0.431 | 0 | 41.11 |
| IL27 | Stat1 | 1.368 | 0.383 | <0.001 | 0.461 | 0.32 | 0.149 | 55.9 |
| IL27 | IL22 | 0.391 | 0.488 | 0.423 | 1.794 | 0.571 | 0.002 | 45.54 |
| IL27ra | Csf2 | -1.05 | 0.409 | 0.01 | 2.238 | 0.447 | 0 | 44.97 |
| IL27ra | Tbx21 | -1.021 | 0.405 | 0.012 | 2.404 | 0.499 | 0 | 45.41 |
| IL27ra | IL13 | 0.004 | 0.247 | 0.987 | 0.407 | 0.262 | 0.12 | 83.6 |
| IL27ra | Ccl7 | 0.069 | 0.286 | 0.809 | 1.153 | 0.295 | 0 | 67.24 |
| IL27ra | Ccr2 | 0.365 | 0.309 | 0.237 | -0.499 | 0.306 | 0.104 | 83.42 |
| IL27ra | Ccr3 | 0.373 | 0.334 | 0.265 | -0.446 | 0.333 | 0.18 | 84.35 |
| IL27ra | Ccr5 | -0.253 | 0.292 | 0.386 | 1.241 | 0.326 | 0 | 67.18 |
| IL27ra | Gata3 | 0.356 | 0.29 | 0.219 | -0.55 | 0.293 | 0.061 | 82.48 |
| IL27ra | Irf1 | -0.585 | 0.386 | 0.13 | 1.863 | 0.434 | 0 | 52.97 |
| IL27ra | Socs1 | -0.629 | 0.406 | 0.121 | 2.195 | 0.476 | 0 | 44.97 |
| IL27ra | Stat1 | -0.453 | 0.315 | 0.15 | 1.199 | 0.33 | 0 | 69.55 |
| IL27ra | IL22 | -0.275 | 0.383 | 0.472 | 2.212 | 0.525 | 0 | 45.67 |
| Csf2 | Tbx21 | 1.438 | 0.475 | 0.002 | 1.656 | 0.565 | 0.003 | 41.71 |
| Csf2 | IL13 | 2.197 | 0.498 | <0.001 | -0.603 | 0.354 | 0.088 | 49.87 |
| Csf2 | Ccl7 | 1.466 | 0.386 | <0.001 | 0.708 | 0.338 | 0.036 | 48.34 |
| Csf2 | Ccr2 | 1.984 | 0.417 | <0.001 | -0.831 | 0.38 | 0.029 | 46.86 |
| Csf2 | Ccr3 | 1.868 | 0.386 | <0.001 | -0.643 | 0.337 | 0.056 | 48.8 |
| Csf2 | Ccr5 | 1.648 | 0.464 | <0.001 | 0.194 | 0.401 | 0.628 | 52.59 |
| Csf2 | Gata3 | 2.597 | 0.544 | <0.001 | -1.481 | 0.436 | 0.001 | 36.55 |
| Csf2 | Irf1 | 1.138 | 0.49 | 0.02 | 0.81 | 0.457 | 0.076 | 49.44 |
| Csf2 | Socs1 | 0.58 | 0.594 | 0.329 | 1.404 | 0.617 | 0.023 | 46.64 |
| Csf2 | Stat1 | 2.06 | 0.602 | 0.001 | -0.298 | 0.448 | 0.506 | 52.37 |
| Csf2 | IL22 | 0.538 | 0.54 | 0.319 | 1.568 | 0.646 | 0.015 | 45.17 |
| Tbx21 | IL13 | 2.153 | 0.525 | <0.001 | 0.468 | 0.357 | 0.189 | 51.14 |
| Tbx21 | Ccl7 | 1.795 | 0.493 | <0.001 | 0.725 | 0.333 | 0.03 | 47.93 |
| Tbx21 | Ccr2 | 2.078 | 0.491 | <0.001 | -0.565 | 0.313 | 0.071 | 49.3 |
| Tbx21 | Ccr3 | 2.07 | 0.486 | <0.001 | -0.571 | 0.337 | 0.09 | 49.67 |
| Tbx21 | Ccr5 | 1.791 | 0.487 | <0.001 | 0.753 | 0.379 | 0.047 | 48.73 |
| Tbx21 | Gata3 | 1.91 | 0.466 | <0.001 | -0.375 | 0.348 | 0.281 | 51.74 |
| Tbx21 | Irf1 | 1.624 | 0.534 | 0.002 | 1.049 | 0.362 | 0.004 | 42.78 |
| Tbx21 | Socs1 | 1.441 | 0.563 | 0.011 | 1.383 | 0.425 | 0.001 | 39.12 |
| Tbx21 | Stat1 | 1.983 | 0.533 | <0.001 | 0.712 | 0.34 | 0.036 | 48.11 |
| Tbx21 | IL22 | 1.341 | 0.532 | 0.012 | 1.541 | 0.486 | 0.002 | 38.09 |
| IL13 | Ccl7 | -0.259 | 0.332 | 0.435 | 1.311 | 0.371 | 0 | 66.69 |
| IL13 | Ccr2 | 0.576 | 0.278 | 0.038 | -0.49 | 0.276 | 0.076 | 80.18 |
| IL13 | Ccr3 | 0.568 | 0.285 | 0.047 | -0.419 | 0.284 | 0.139 | 81.25 |
| IL13 | Ccr5 | -1.603 | 0.568 | 0.005 | 2.622 | 0.667 | 0 | 58.04 |
| IL13 | Gata3 | 0.97 | 0.33 | 0.003 | -0.969 | 0.339 | 0.004 | 74.3 |
| IL13 | Irf1 | 0.125 | 0.302 | 0.677 | 1.565 | 0.362 | 0 | 55.36 |
| IL13 | Socs1 | 0.112 | 0.3 | 0.709 | 1.88 | 0.397 | 0 | 47.46 |
| IL13 | Stat1 | 0.206 | 0.282 | 0.466 | 0.939 | 0.29 | 0.001 | 71.21 |
| IL13 | IL22 | -0.502 | 0.332 | 0.13 | 2.454 | 0.554 | 0 | 43.85 |
| Ccl7 | Ccr2 | 1.147 | 0.299 | <0.001 | -0.218 | 0.274 | 0.425 | 66.66 |
| Ccl7 | Ccr3 | 1.309 | 0.325 | <0.001 | -0.51 | 0.291 | 0.08 | 64.03 |
| Ccl7 | Ccr5 | 0.833 | 0.308 | 0.007 | 0.889 | 0.348 | 0.011 | 59.99 |
| Ccl7 | Gata3 | 1.15 | 0.303 | <0.001 | -0.318 | 0.295 | 0.282 | 66.13 |
| Ccl7 | Irf1 | 0.488 | 0.355 | 0.17 | 1.299 | 0.399 | 0.001 | 53.61 |
| Ccl7 | Socs1 | 0.556 | 0.334 | 0.096 | 1.736 | 0.424 | 0 | 44.74 |
| Ccl7 | Stat1 | 0.868 | 0.335 | 0.01 | 0.548 | 0.325 | 0.092 | 64.36 |
| Ccl7 | IL22 | 0.215 | 0.347 | 0.536 | 1.956 | 0.501 | 0 | 45.81 |
| Ccr2 | Ccr3 | -0.424 | 0.452 | 0.347 | 0.168 | 0.447 | 0.707 | 84.72 |
| Ccr2 | Ccr5 | -1.588 | 0.552 | 0.004 | 2.093 | 0.523 | 0 | 54.75 |
| Ccr2 | Gata3 | -0.006 | 0.384 | 0.987 | -0.355 | 0.389 | 0.362 | 84.03 |
| Ccr2 | Irf1 | 0.151 | 0.312 | 0.629 | 1.642 | 0.367 | 0 | 55.3 |
| Ccr2 | Socs1 | 0.117 | 0.317 | 0.713 | 1.928 | 0.4 | 0 | 47.47 |
| Ccr2 | Stat1 | -0.134 | 0.263 | 0.61 | 0.972 | 0.288 | 0.001 | 71.49 |
| Ccr2 | IL22 | -0.042 | 0.357 | 0.907 | 2.084 | 0.46 | 0 | 46.18 |
| Ccr3 | Ccr5 | -0.918 | 0.409 | 0.025 | 1.555 | 0.397 | 0 | 61.32 |
| Ccr3 | Gata3 | 0.093 | 0.322 | 0.773 | -0.424 | 0.332 | 0.201 | 83.94 |
| Ccr3 | Irf1 | -0.116 | 0.288 | 0.686 | 1.598 | 0.358 | 0 | 55.38 |
| Ccr3 | Socs1 | 0.084 | 0.304 | 0.783 | 1.924 | 0.404 | 0 | 47.53 |
| Ccr3 | Stat1 | -0.259 | 0.26 | 0.319 | 1.008 | 0.287 | 0 | 70.75 |
| Ccr3 | IL22 | -0.015 | 0.35 | 0.966 | 2.083 | 0.458 | 0 | 46.2 |
| Ccr5 | Gata3 | 2.022 | 0.467 | <0.001 | -1.461 | 0.414 | 0 | 51.18 |
| Ccr5 | Irf1 | 0.977 | 0.345 | 0.005 | 1.389 | 0.357 | 0 | 46.8 |
| Ccr5 | Socs1 | 0.793 | 0.349 | 0.023 | 1.649 | 0.399 | 0 | 42.08 |
| Ccr5 | Stat1 | 1.056 | 0.319 | 0.001 | 0.84 | 0.295 | 0.004 | 58.73 |
| Ccr5 | IL22 | 0.387 | 0.394 | 0.327 | 1.818 | 0.502 | 0 | 45.22 |
| Gata3 | Irf1 | -0.323 | 0.307 | 0.293 | 1.591 | 0.361 | 0 | 54.42 |
| Gata3 | Socs1 | -0.485 | 0.331 | 0.142 | 1.972 | 0.41 | 0 | 45.33 |
| Gata3 | Stat1 | -0.484 | 0.279 | 0.083 | 1.055 | 0.299 | 0 | 68.59 |
| Gata3 | IL22 | -0.53 | 0.324 | 0.102 | 2.3 | 0.539 | 0 | 43.35 |
| Irf1 | Socs1 | -0.38 | 0.787 | 0.629 | 2.259 | 0.847 | 0.008 | 47.37 |
| Irf1 | Stat1 | 4.111 | 0.966 | <0.001 | -2.582 | 0.874 | 0.003 | 44.36 |
| Irf1 | IL22 | 0.291 | 0.514 | 0.572 | 1.819 | 0.641 | 0.005 | 45.87 |
| Socs1 | Stat1 | 3.276 | 0.787 | <0.001 | -1.474 | 0.652 | 0.024 | 41.21 |
| Socs1 | IL22 | 0.955 | 0.623 | 0.125 | 1.222 | 0.675 | 0.071 | 43.82 |
| Stat1 | IL22 | -0.664 | 0.44 | 0.131 | 2.753 | 0.694 | 0 | 43.84 |

Using qRT-PCR data only (Table S1, “qRT-PCR;” univariate results shown in Table S3), this table shows the estimated coefficient (Coef), standard error (SE), P values (P), and Akaike information criterion (AIC) for all possible pairs of predictors as calculated by logistic regression. The p value is a test as to whether relative expression levels of both selected genes have any effect on survival.
