# Supplementary material for: Development of Functional and Molecular Correlates of Vaccine-Induced Protection for a Model Intracellular Pathogen, F. tularensis LVS
Source: PLoS Pathog. 2012 Jan 19;8(1):e1002494. doi: 10.1371/journal.ppat.1002494 (PMC3262015; doi:10.1371/journal.ppat.1002494)
Supplement: Text S1 — Optimization of conditions for analyses. (DOC) [file ppat.1002494.s011.doc]

**Supporting Information, Text**

**Optimization of conditions for analyses**

**Additional Materials and Methods**

*Cytokine and nitrite measurements.* Supernatants recovered from *in vitro* co-cultures were assayed using a standard sandwich ELISAs, according to the manufacturer’s instructions (BD Pharmingen, San Diego, CA). The absorbance was read at 405 nm on a VersaMax tunable microplate reader with a reference wavelength of 630 nm (Molecular Devices, Sunnyvale, CA). Cytokines were quantitated by comparison to recombinant standard proteins (BD Pharmingen) using four-parameter fit regression in the SOFTmax Pro ELISA analysis software (Molecular Devices). Antibody pairs and standards were purchased from BD Pharmigen. NO was estimated in culture supernatants using the Griess reaction. Samples of supernatants were incubated with an equal volume of commercial Griess reagent (Sigma-Aldrich, St. Louis, MO) and absorbance was measured at 490 nm. Nitrite (NO_2_) was measured by comparison to serially diluted NaNO_2_ as a standard using four-parameter fit regression .

*In vitro cell purification.* A MACS Midi system and anti-Thy1.2 beads (Miltenyi Biotec, Auburn, CA) were used to enrich T cell subpopulations, according to standard protocols. Single-cell suspensions of splenocytes obtained from either vaccinated or naive mice were treated with the appropriate amount of magnetic beads according the manufacturer’ instructions. The composition and relative purity of the resulting enriched cells were assessed by multiparameter flow cytometry.

**Supporting Information Results and Discussion**

Previous studies with the *in vitro* co-culture system reliably reflected the activities of T cell subpopulations and major mechanisms that are necessary for successful *in vivo* vaccination with the leading *Francisella* vaccine candidate, LVS . In those studies, control of intramacrophage LVS replication was used as the endpoint, but control of infection is a dynamic process. Here, our goal was to evaluate the ability of the *in vitro* co-culture approach to discriminate between several qualitatively different *Francisella* vaccine candidates, and if so take further advantage of the *in vitro* model to identify T cell-related correlates associated with limiting intracellular *Francisella* replication. Therefore, to determine informative and optimal time points for analyses, host cells recovered from co-cultures were evaluated for cell composition, cell viability, and RNA integrity over time, in relationship to control of intramacrophage LVS growth. Wild-type (WT) C57BL/6J mice were vaccinated ID with *F. tularensis* LVS or with PBS (throughout, naive control), and sacrificed 6 – 8 weeks after immunization. Splenocytes from vaccinated or naive mice were co-cultured with LVS-infected macrophages for 1, 2 or 3 days; supernatants were harvested for analyses of secreted mediators, non-adherent cells were recovered for analyses of cell subpopulations and preparation of mRNA, and adherent cells recovered to determine bacterial replication as well as for mRNA preparation. Consistent with previous data , differences between bacterial growth in co-cultures containing naive splenocytes compared to those with LVS-immune splenocytes were significant by two days, and most evident after three days of co-culture (Figure S1). However, a ~1.5 log difference between naive and LVS-vaccinated mice was found after only two days, confirming that significant vaccine-induced T cell activity can readily be detected by this time point. Non-adherent splenocytes that were recovered from co-cultures were then characterized by flow cytometry. Total numbers of viable recovered cells are presented for each group, followed by the proportion of each indicated cell subpopulation (Table S1). Total cell numbers decreased with increasing time in culture; further, cell viability decreased from about 80% to about 50 – 55%. However, the proportion of T cells increased, while B cells decreased, and natural killer, dendritic cells, neutrophils and macrophages did not persist (Table S1). The latter trends were especially evident in LVS-vaccinated splenocytes, implying that the presence of LVS in culture stimulates previously primed T lymphocytes.

Total RNA was purified from the non-adherent splenocytes and analyzed to assess nucleic acid quality. RNA quality degraded progressively over three days of culture, with the RNA integrity number (RIN) after three days of culture ranging from 5.3 to 6.8 for naive and LVS-vaccinated splenocytes, respectively. This was considered unacceptable for downstream experiments, such as RT-PCR. At day 2, however, the RIN ranged from 8.4 to 8.7; although lower than values determined for RNA prepared on day 0 or day 1, which were consistently above 9.0, this quality was considered excellent and thus acceptable, and used as a quality control reference for future experiments.

The lack of macrophages detected among the non-adherent cells recovered from co-cultures by day 2 (Table S1) indicated little carryover of adherent LVS-infected macrophages that might impact the gene expression profiles of the recovered splenocytes. To further evaluate the relative contributions of adherent LVS-infected macrophages and non-adherent splenocytes to the levels of mRNA and subsequent secretion of cytokines, IFN-γ, TNF-α, IL-12 p40, and IL-6 mRNA levels and protein production were directly compared. As shown in Figure S2, gene expression of cytokine mRNA analyzed by qRT-PCR on day 2 clearly confirmed that TNF-α, IL-12 p40, and IL-6 mRNA were present not only in non-adherent cells, but also in macrophages responding to LVS infection. The levels of expression were modulated, in both types of cells, by the addition of splenocytes, especially those from LVS-vaccinated mice. Elevated levels of IFN-γ mRNA were present only in the splenocyte fraction, and only detected at very low levels in the infected macrophage fraction when co-cultured with immune splenocytes; this was likely due to inadvertent recovery of lymphocytes in the macrophage fraction. Consistent with mRNA results, analyses of protein present in supernatants by ELISA demonstrated that LVS-infected macrophages alone secreted TNF-α, IL-12 p40, and IL-6 (Figure S3). Co-culture of LVS-infected macrophages with splenocytes, especially those from LVS-vaccinated mice, increased the secretion of all three of these cytokines; however, substantial amounts of IFN-γ were only secreted when LVS-immune splenocytes were co-cultured with LVS-infected macrophages. Cytokine proteins were detected in co-culture supernatants by day 2, although quantities of all four cytokines accumulated and increased through day 3 (Figure S3). Collectively, therefore, two days of co-culture was chosen as ideal time point to analyze gene expression, since differences between co-cultures containing naive and LVS-vaccinated splenocytes were detected in terms of both bacterial control and cytokine production, and the quality of RNA prepared from recovered splenocytes remained excellent.

We then performed initial studies to examine whether these conditions faithfully discriminated between vaccination status when expanded analyses of gene expression in recovered cells were performed. C57BL/6J mice were vaccinated with 10^4^ LVS ID, and splenocytes were prepared from naive or vaccinated (LVS-immune) mice, co-cultured with LVS-infected macrophages for two days, and non-adherent cells recovered to prepare mRNA. The relative mRNA expression of genes of immunologic interest in naive or LVS-immune splenocytes was compared by Profiler PCR Th1-Th2-Th3 arrays that included 84 immunologically-related genes. Here, gene amplification was semi-quantitatively calculated by normalization to the average from five housekeeping genes included in the assay, and the specificity of the amplicons was assessed by evaluation of the dissociation profiles. A comparative analysis between naive and LVS-treated splenocytes revealed differential gene expression between LVS-immune and naive cells, including both up- and down-regulation, of several genes (Table S2; experiments 1, 2, and 3). In replicate experiments, 12 genes (14%) were consistently increased two fold or more in LVS-immune splenocytes compared to naive splenocytes, 6 genes (7%) were decreased 0.5 fold or more, and 66 genes (78%) were not considered different. Results in non-adherent splenocytes using the commercial arrays agreed well with qRT-PCR results obtained using independent primers and probes for selected genes, and increased confidence in the screening approach (see Table S2). Also of note, a number of genes that were up-regulated in cells from LVS-immune mice exhibited readily detectable increases; however, most genes that were down-regulated exhibited less dramatic decreases, and as a result appeared to be less useful in distinguishing between LVS-immune and naive cells.

Previous results indicated that intramacrophage control of LVS growth in the co-culture system was primarily a function of T lymphocytes . Nonetheless, inclusion of non-T cells during co-cultures may optimize or influence T cell activities. To consider this aspect in the context of the present studies, C57BL/6J mice were vaccinated by treatment with 10^4^ LVS ID; 4 – 6 weeks later enriched T cells, comprised of ~ 95% TCRβ^+^ Thy1.2^+^ cells as determined by multiparameter flow cytometry, were prepared from splenocytes from naive and LVS-immune mice. Enriched T cells were co-cultured with LVS-infected macrophages, and non-adherent cells recovered to prepare mRNA for similar analyses. As might be expected, studies using enriched T cells instead of whole splenocytes yielded qualitatively similar results, although there were differences in the relative increase or decrease in expression of individual T cell-related genes (data not shown). We therefore elected to use whole splenocyte preparations in subsequent experiments, in order to permit interactions between all available responding cells, as well as minimize introduction of bias during enrichment.

**References for Supporting Information text**

1. Green LC, Wagner A, Glogowski J, Skipper PL, Wishnok JS, et al. (1982) Analysis of nitrate, nitrite, and [15N]nitrate in biological fluids. Anal Biochem 126: 131-138.

2. Cowley SC, Elkins KL (2003) Multiple T cell subsets control *Francisella tularensis* LVS intracellular growth without stimulation through macrophage interferon gamma receptors. J Exp Med 198: 379-389.

3. Cowley SC, Hamilton E, Frelinger JA, Su J, Forman J, et al. (2005) CD4^-^CD8^-^ T cells control intracellular bacterial infections both *in vitro* and *in vivo*. J Exp Med 202: 309-319.

4. Elkins KL, Cowley SC, Conlan JW (2011) Measurement of macrophage-mediated killing of intracellular bacteria, including *Francisella* and *Mycobacteria*. Curr Protoc Immunol 93: 14.25.11-14.25.13.

5. Collazo CM, Meierovics AI, De Pascalis R, Wu TH, Lyons CR, et al. (2009) T cells from lungs and livers of *Francisella tularensis*-immune mice control the growth of intracellular bacteria. Infect Immun 77: 2010-2021.

6. Elkins KL, Cooper A, Colombini SM, Cowley SC, Kieffer TL (2002) In vivo clearance of an intracellular bacterium, *Francisella tularensis* LVS, is dependent on the p40 subunit of Interleukin-12 (IL-12) but not on IL-12 p70. Infect Immun 70: 1936-1948.

7. Bosio CM, Elkins KL (2001) Susceptibility to secondary *Francisella tularensis* LVS infection in B cell deficient mice is associated with neutrophilia but not with defects in specific T cell mediated immunity. Infect Immun 69: 194-203.
